# Supplementary material for: Complete Genome Sequences and Genome-Wide Characterization of Trichoderma Biocontrol Agents Provide New Insights into their Evolution and Variation in Genome Organization, Sexual Development, and Fungal-Plant Interactions
Source: Microbiol Spectr. 2021 Dec 15;9(3):e00663-21. doi: 10.1128/Spectrum.00663-21 (PMC8672877; doi:10.1128/Spectrum.00663-21)
Supplement: SUPPLEMENTAL FILE 1 — Supplemental material. Download SPECTRUM00663-21_Supp_1_seq7.pdf, PDF file, 3.5 MB [file spectrum00663-21_supp_1_seq7.pdf]

# Supporting Information

**Complete Genome Sequences and Genome-Wide Characterization of *Trichoderma* Biocontrol Agents Provide New Insights into Evolution and Variation in Genome Organization, Sexual Development and Fungal-Plant Interactions**

Wan-Chen Li[1], Ting-Chan Lin[1,2], Chia-Ling Chen[1], Hou-Cheng Liu[1], Hisn-Nan Lin[1], Ju-Lan Chao[1], Cheng-Hsilin Hsieh[1], Hui-Fang Ni[3], Ruey-Shyang Chen[2,#], Ting-Fang Wang[1,2,#]

[1]Institute of Molecular Biology, Academia Sinica, Taipei 115, Taiwan
[2]Department of Biochemical Science and Technology, National Chiayi University, Chiayi 600, Taiwan
[3]Department of Plant Protection, Chiayi Agricultural Experiment Station, Council of Agriculture, Chiayi 600, Taiwan

#Corresponding authors:
RSC, email: rschen@mail.ncyu.edu.tw
TFW, email: tfwang@gate.sinica.edu.tw

The supplemental information (*SI*) file includes 9 supplemental tables and 24 supplemental figures. Fifteen supplemental datasets (DS) in Excel format are also provided separately.

**Other authors' email addresses:**

| | |
|---|---|
| Wan-Chen Li | wanwan9121@hotmail.com |
| Ting-Chan Lin: | jacky1998711@gmail.com |
| Chia-Ling Chen | chialing.chen1118@gmail.com |
| Hou-Cheng Liu | hc666@gate.sinica.edu.tw |
| Ju-Lan Chao | julan@gate.sinica.edu.tw |
| Hsin-Nan Lin | arith@gate.sinica.edu.tw |
| Cheng-Hsilin Hsieh | imbhch@gate.sinica.edu.tw |
| Hui-Fang Ni | hfni@tari.gov.tw |

Table S1. List of all *Trichoderma* strains analyzed in this study

| Strain name | References |
|---|---|
| *Trichoderma reesei* QM6a | (1) |
| *Trichoderma reesei* CBS1-2 | (2) |
| *Trichoderma reesei* CBS1-2 | (2) |
| *Trichoderma virens* Gv29-8 | NCBI Bioproject accession PRJNA700774, GenBank: CP071107-CP071114 |
| *Trichoderma virens* FT-333 | NCBI Bioproject accession PRJNA700774, GenBank: CP071115-CP071122 |
| *Trichoderma asperellum* FT-101 | NCBI Bioproject accession PRJNA700774, GenBank: CP084943-CP084950 |
| *Trichoderma atroviride* P1 | NCBI Bioproject accession PRJNA700774, GenBank: CP084935-CP084942 |

Table S2. Summary of authentic transposable elements[1] in the seven near-complete *Trichoderma* genome sequences

| Species | | *T. reesei* | *T. reesei* | *T. reesei* | *T. virens* | *T. virens* | *T. asperellum* | *T. atroviride* |
|---|---|---|---|---|---|---|---|---|
| Strain | | CBS1-1 | CBS1-2 | QM6a | Gv29-8 | FT-333 | FT-101 | P1 |
| Overall | | 62 | 62 | 70 | 93 | 94 | 92 | 78 |
| Class I retrotransposons | Tad1-LINE | 0 | 0 | 0 | 2 | 8 | 1 | 1 |
| | RI-LINE | 0 | 0 | 0 | 7 | 1 | 4 | 8 |
| | Jockey-LINE | 11 | 11 | 4 | 6 | 9 | 10 | 10 |
| | other LINEs | 11 | 11 | 14 | 4 | 4 | 1 | 5 |
| | Copia-LTR | 5 | 5 | 8 | 4 | 4 | 4 | 3 |
| | Gypsy-LTR | 2 | 2 | 10 | 11 | 19 | 43 | 18 |
| | other LTRs | 6 | 6 | 4 | 8 | 7 | 7 | 9 |
| Class II transposons | CMC-EnSpm | 4 | 4 | 6 | 2 | 2 | 2 | 6 |
| | MULE-MuDR | 1 | 1 | 0 | 28 | 18 | 6 | 6 |
| | hAT-Charlie | 17 | 17 | 21 | 2 | 1 | 1 | 1 |
| | TcMar -Ant1 | 0 | 0 | 0 | 5 | 8 | 2 | 1 |
| | PIF-Harbinger-like | 0 | 0 | 0 | 1 | 0 | 1 | 0 |
| | Others | 5 | 5 | 3 | 13 | 13 | 10 | 10 |

1. RepeatMasker (version 4.0.6) was applied to identify the authentic transposable elements, with preliminary RepeatMasker data being filtered using two parameters (length ≥140, Smith–Waterman local similarity scores ≥450). Using the same two parameters, we were able to accurately identify the location of five different Ty elements in the 16 chromosomes of the budding yeast *S. cerevisiae* (1).

Table S3. The location of predicted centromeres in CBS1-2, Gv29-8, FT-101 and P1

| Chromosome | QM6a | CBS1-1 | CBS1-2 |
|---|---|---|---|
| ChI | 3100001-3305000 (~205kb) | 3184500- 3359500 (~175kb) | 3184500- 3359500 (~175kb) |
| ChII | 1940001-2105000 (~165kb) | 1892000-2063500 (~172kb) | 1892000-2063500 (~172kb) |
| ChIII | 1745001-1905000 (~160kb) | 1677500-1830500 (~153kb) | 1677500-1830500 (~153kb) |
| ChIV | 1480001-1660000 (~180kb) | 1453500- 1617500 (~164kb) | 1453500- 1617500 (~164kb) |
| ChV | 1165001-1330000 (~165kb) | 1102500-1259500 (~157kb) | 1102500-1259500 (~157kb) |
| ChVI | 1825001-2035000 (~210kb) | 1636500-1810000 (~174kb) | 1637000-1810500 (~174kb) |
| CVII | 1740001-1920000 (~180kb) | 1858000-2018500 (~161kb) | 1858000-2018500 (~161kb) |

| Chromosome | Gv29-8 | FT-333 | FT-101 | P1 |
|---|---|---|---|---|
| ChI | 3568001-3671500 (~103 kb) | 3733001-3835000 (~102 kb) | 4246001-4351000 (~105 kb) | 3038001-3130500 (~92 kb) |
| ChII | 4049001-4156500 (~107 kb) | 4039501-4144500 (~105 kb) | 3025501-3130500 (~105 kb) | 3084001-3208000 (~124 kb) |
| ChIII | 4163501-4275500 (~112 kb) | 4184001-4261000 (~77 kb) | 1795501-1897500 (~102 kb) | 1919001-2039000 (~120 kb) |
| ChIV | 2098501-2211500 (~113 kb) | 2181501-2285500 (~104 kb) | 3176001-3297500 (~121 kb) | 2118501-2220000 (~101 kb) |
| ChV | 3087001-3195500 (~108 kb) | 3219501-3324500 (~105 kb) | 2775001-2874000 (~99 kb) | 1969501-2113000 (~143 kb) |
| ChVI | 1910501-1992500 (~82 kb) | 1931001-2031000 (~100 kb) | 2199001-2280000 (~81 kb) | 1227501-1348000 (~120 kb) |
| CVII | 2680501-2767000 (~86 kb) | 2688001-2775000 (~87 kb) | 1580001-1692000 (~112 kb) | 1611001-1704000 (~93 kb) |

Table S4. Numbers of annotated transcription factors encoded by the near-complete genomes of the seven *Trichoderma* spp.

| InterPro | Description | CBS1-1 | CBS1-2 | QM6a | Gv29-8 | FT-333 | FT-101 | P1 |
|---|---|---|---|---|---|---|---|---|
| 000967 | NF-X1-type zinc finger | 2 | 2 | 2 | 0 | 0 | 2 | 2 |
| 006856 | Mating-type protein MAT alpha-1 HMG-Box | 1 | 0 | 0 | 0 | 1 | 1 | 0 |
| 018501 | DDT domain | 1 | 1 | 1 | 1 | 1 | 1 | 1 |
| 007196 | CCR4-Not complex component | 1 | 1 | 1 | 1 | 1 | 1 | 1 |
| 007396 | Putative FMN-binding domain | 1 | 1 | 1 | 3 | 3 | 1 | 1 |
| 004595 | TFIIH C1-like domain | 1 | 1 | 1 | 1 | 0 | 1 | 0 |
| 004181 | MIZ zinc finger | 4 | 4 | 4 | 3 | 3 | 3 | 3 |
| 000818 | TEA/ATTS domain family | 1 | 1 | 1 | 1 | 1 | 1 | 1 |
| 001387 | Helix-turn-helix | 1 | 1 | 1 | 1 | 1 | 1 | 1 |
| 001289 | CCAAT-binding TF (CBF-B/NF-YA) subunit B | 1 | 1 | 1 | 1 | 1 | 1 | 1 |
| 003120 | STE-like TF | 1 | 1 | 1 | 1 | 1 | 1 | 1 |
| 003150 | RFX DNA-binding domain | 2 | 2 | 2 | 2 | 2 | 2 | 2 |
| 004198 | Zinc finger | 1 | 1 | 1 | 1 | 1 | 1 | 1 |
| 007604 | CP2 TF | 1 | 1 | 1 | 1 | 1 | 2 | 1 |
| 008895 | YL1 nuclear protein | 1 | 1 | 1 | 1 | 1 | 1 | 2 |
| 010770 | SGT1 protein | 1 | 1 | 1 | 1 | 1 | 1 | 1 |
| 018004 | KilA-N domain | 5 | 6 | 4 | 4 | 2 | 4 | 4 |
| 024061 | NDT80/PhoG-like DNA-binding family | 4 | 6 | 4 | 3 | 3 | 3 | 4 |
| 003656 | BED zinc finger | 1 | 0 | 0 | 2 | 1 | 1 | 1 |
| 018060 | Bacterial regulatory HTH proteins | 1 | 1 | 1 | 2 | 1 | 4 | 4 |
| 005011 | SART-1 family | 1 | 1 | 1 | 1 | 1 | 1 | 1 |
| 002100 | SRF-type TF (DNA-binding and dimerization domain) | 2 | 1 | 2 | 2 | 2 | 2 | 3 |
| 010666 | GRF zinc finger | 2 | 2 | 2 | 1 | 1 | 3 | 2 |
| 000232 | HSF-type DNA-binding | 4 | 3 | 5 | 3 | 3 | 4 | 4 |
| 001766 | Fork head domain | 4 | 4 | 4 | 5 | 5 | 4 | 4 |
| 001356 | Homeobox domain | 9 | 10 | 12 | 9 | 9 | 12 | 11 |
| 000679 | GATA zinc finger | 8 | 9 | 7 | 7 | 6 | 7 | 8 |
| 013767 | PAS fold | 1 | 1 | 1 | 0 | 0 | 0 | 0 |
| 001878 | Zinc knuckle (CCHC) | 13 | 13 | 11 | 10 | 10 | 11 | 14 |
| 04827 | Basic region leucine zipper 2 | 27 | 28 | 28 | 27 | 28 | 28 | 26 |
| 00788 | Helix-turn-helix | 0 | 0 | 1 | 1 | 1 | 1 | 1 |
| 011598 | Helix-loop-helix DNA-binding domain | 10 | 10 | 10 | 10 | 9 | 10 | 10 |
| 001005 | Myb-like DNA-binding domain | 18 | 17 | 17 | 17 | 15 | 16 | 17 |
| 004827 | bZIP TF 1 | 27 | 28 | 28 | 27 | 28 | 28 | 26 |
| 007087 | Zinc finger (C2H2) | 86 | 83 | 85 | 81 | 73 | 90 | 85 |
| 001138 | Fungal Zn(2)-Cys(6) binuclear cluster domain | 276 | 291 | 267 | 293 | 227 | 384 | 374 |
| 007219 | Fungal-specific TF domain | 171 | 176 | 170 | 215 | 197 | 248 | 225 |

Table S5. Numbers of SM-BGCs and CAZ-BGCs revealed by antiSMASH

| | Chromosome | CBS1-1 | CBS1-2 | QM6a | Gv29-8 | FT-333 | FT-101 | P1 |
|---|---|---|---|---|---|---|---|---|
| SM-BGC[1] | ChI | 5 | 5 | 5 | 11 | 10 | 9 | 8 |
| | ChII | 8 | 7 | 7 | 8 | 8 | 7 | 8 |
| | ChIII | 4 | 4 | 4 | 13 | 13 | 7 | 5 |
| | ChIV | 2 | 3 | 3 | 5 | 3 | 4 | 7 |
| | ChV | 3 | 3 | 3 | 4 | 6 | 6 | 9 |
| | ChVI | 7 | 7 | 7 | 11 | 10 | 9 | 2 |
| | CVII | 3 | 3 | 2 | 6 | 7 | 10 | 6 |
| | Total | 32 | 32 | 31 | 58 | 57 | 54 | 45 |
| CAZ-BGC[2] | ChI | 5 | 5 | 4 | 5 | 3 | 6 | 6 |
| | ChII | 7 | 7 | 5 | 5 | 3 | 2 | 4 |
| | ChIII | 3 | 3 | 2 | 4 | 3 | 4 | 5 |
| | ChIV | 6 | 6 | 7 | 7 | 8 | 3 | 3 |
| | ChV | 3 | 3 | 3 | 7 | 2 | 5 | 3 |
| | ChVI | 3 | 3 | 3 | 4 | 4 | 7 | 6 |
| | CVII | 4 | 4 | 5 | 3 | 3 | 4 | 6 |
| | Total | 31 | 31 | 29 | 35 | 26 | 31 | 33 |

1. SM-BGC: secondary metabolite biosynthetic gene cluster.
2. CAZ-BGC: carbohydrate-active enzyme (CAZyme) gene cluster.

Table S6. Comparative analyses of seven previously characterized SM-BGCs. OrthoVenn2 (https://orthovenn2.bioinfotoolkits.net/home) was applied to search (E = ≤ $10^{-5}$) for orthologs of SM-BGCs. Gene IDs and their chromosome number (in brackets) are indicated.

| Siderophore (SID)-BGCs | | | | | |
|---|---|---|---|---|---|
| JGI ID | CBS1-2 | Gv29-8 | P1 | FT-101 | Annotation |
| | SM-BGC 4.2 | SM-BGC 6.4 | SM-BGC 7.3 | SM-BGC 6.5 | |
| Tr-67189 | TRC2_005918 (IV) | TrV_010298 (VI) | TrAt_012594 (VII) | TrA_010145 (VI) | *pks4* NRPS |
| Tr-66999 | TRC2_005919 (IV) | TrV_010299 (VI) | TrAt_012595 (VII) | TrA_010144 (VI) | NRPS-like protein |
| Tr-67109 | TRC2_005920 (IV) | TrV_010300 (VI) | TrAt_012596 (VII) | TrA_010143 (VI) | O-acetyltransferase *sat14* |
| Tr-67026 | TRC2_005921 (IV) | TrV_010301 (VI) | TrAt_012597 (VII) | TrA_010142 (VI) | Siderochrome iron transporter 1 |
| Tr-5206 | TRC2_005922 (IV) | TrV_010302 (VI) | TrAt_012598 (VII) | TrA_010141 (VI) | Oxidoreductase |
| Tr-110499 | TRC2_005923 (IV) | TrV_004842 (III) | TrAt_012599 (VII) | TrA_010139 (VI) | ABC transporter |
| Ferrichrome (FRC)-BGCs | | | | | |
| JGI ID | CBS1-2 | Gv29-8 | P1 | FT-101 | Annotation |
| | SM-BGC 2.2 | SM-BGC 7.5 | SM-BGC 2.3 | SM-BGC 4.3 | |
| Tr-69972 | TRC2_002088 (II) | TrV_011144 (VII) | TrAt_002779 (II) | TrA_006545 (IV) | TF |
| Tr-23368 | TRC2_002089 (II) | TrV_011145 (VII) | TrAt_002782 (II) | TrA_006547 (IV) | PAK-GC kinase *sid1* |
| - | TRC2_002090 (II) | - | - | - | Hypothetical protein |
| Tr-69946 | TRC2_002091 (II) | TrV_011146 (VII) | TrAt_002784 (II) | TrA_006548 (IV) | NRPS |
| Tr-23367 | TRC2_002092 (II) | TrV_011147 (VII) | TrAt_002785 (II) | TrA_006549 (IV) | Oxidoreductase |
| Tr-52375 | TRC2_002093 (II) | TrV_011148 (VII) | TrAt_002786 (II) | TrA_006551 (IV) | Aldehyde dehydrogenase |
| Conidial green pigment (CGP)-BGCs | | | | | |
| JGI ID | CBS1-2 | Gv29-8 | P1 | FT-101 | Annotation |
| | SM-BGC 4.3 | SM-BGC 6.10 | SM-BGC 5.9 | SM-BGC 6.9 | |
| Tr-37950 | TRC2_006733 (IV) | TrV_004745 (II) | - | - | Cytochrome P450 |
| Tr-70127 | TRC2_006734 (IV) | TrV_004747 (II) | - | - | RTA-like protein |
| Tr-112115 | TRC2_006735 (IV) | TrV_004748 (II) | - | - | MFS transporter |
| Tr-112114 | TRC2_006736 (IV) | - | - | - | Hypothetical protein |
| Tr-124079 | TRC2_006737 (IV) | TrV_010890 (VI) | TrAt_010562 (V) | TrA_010757 (VI) | Multicopper oxidase |
| Tr-52476 | TRC2_006738 (IV) | TrV_010891 (VI) | TrAt_010561 (V) | TrA_010756 (VI) | Hypothetical protein |
| Tr-82208 | TRC2_006739 (IV) | TrV_010892 (VI) | TrAt_010560 (V) | TrA_010755 (VI) | Polyketide synthase |
| Tr-112105 | TRC2_006740 (IV) | TrV_009953 (VI) | TrAt_011901 (VI) | - | Hypothetical protein |
| Sorbicillinoid (SOR)-BGCs | | | | | |
| JGI ID | CBS1-2 | Gv29-8 | P1 | FT-101 | Annotation |
| | SM-BGC 5.2 | - | - | - | |
| Tr-53776 | TRC2_007361 (V) | TrV_007389 (IV) | TrAt_007655 (IV) | TrA_011012 (VII) | *usk1* |
| Tr-102492 | TRC2_007362 (V) | TrV_008886 (V) | TrAt_000805 (I) | TrA_001604 (I) | *sor8* |
| Tr-73618 | TRC2_007363 (V) | TrV_000231 (I) | TrAt_002239 (I) | TrA_000207 (I) | *sor1* |
| Tr-73621 | TRC2_007364 (V) | - | TrAt_008990 (IV) | - | *sor2* |

| | | | | | |
|---|---|---|---|---|---|
| Tr-73623 | TRC2_007365 (V) | - | TrAt_008980 (IV) | TrA_006848 (IV) | *sor5* |
| Tr-43701 | TRC2_007366 (V) | - | - | - | *sor4* (MSF) |
| Tr-43701 | TRC2_007367 (V) | - | - | - | *sor4_2* |
| Tr-102497 | TRC2_007368 (V) | TrV_006641 (III) | TrAt_010535 (V) | - | *sor3/ypr2* |
| Tr-73631 | TRC2_007369 (V) | TrV_006377 (III) | - | - | *sor7* |
| Tr-102499 | TRC2_007370 (V) | - | - | - | *sor6/ypr1* |
| Tr-102500 | TRC2_007371 (V) | TrV_008882 (V) | TrAt_000804 (I) | TrA_001605 (I) | |
| Tr-73632 | TRC2_007372 (V) | TrV_006140 (III) | TrAt_004919 (II) | TrA_004330 (II) | *axe1_1* |
| Tr-73638 | TRC2_007373 (V) | TrV_012230 (VII) | TrAt_005240 (III) | TrA_011951 (VII) | |
| Tr-73643 | TRC2_007374 (V) | TrV_012229 (VII) | TrAt_005241 (III) | TrA_011952 (VII) | *cel61a* |
| Trichothecene (TRI)-BGCs | | | | | |
| JGI ID | FT-101 | CBS1-2 | Gv29-8 | P1 | Annotation |
| | SM-BGC 7.3 | - | - | - | |
| Ta-61224 | TrA_010945 (VII) | - | TrV_005642 (III) | TrAt_010304 (V) | Oxidoreductase |
| Ta-30398 | TrA_010946 (VII) | - | TrV_006414 (III) | - | |
| Ta-201111 | TrA_010947 (VII) | - | - | TrAt_010299 (V) | Alcohol dehydrogenase |
| Ta-72517 | TrA_010948 (VII) | TRC2_008174 (VI) | TrV_006398 (III) | TrAt_010298 (V) | |
| Ta-448313 | TrA_010949 (VII) | - | - | - | Trichodiene synthase *tri5* |
| Ta-142955 | TrA_010950 (VII) | TRC2_008175 (VI) | TrV_006397 (III) | TrAt_010297 (V) | |
| Ta-72520 | TrA_010951 (VII) | - | - | TrAt_010295 (V) | |
| Ta-92494 | TrA_010952 (VII) | TRC2_008193 (VI) | TrV_006337 (III) | TrAt_010294 (V) | |
| Viridin (VIR)-BGCs | | | | | |
| JGI ID | Gv29-8 | CBS1-2 | P1 | FT-101 | Annotation |
| | SM-BGC 5.5 | - | - | - | |
| Tv-53366 | TrV_009673 (V) | - | - | TrA_010785 (VII) | *vdn1* CYP |
| Tv-53375 | TrV_009674 (V) | - | - | TrA_010784 (VII) | *vdn2* CYP |
| Tv-230790 | TrV_009675 (V) | - | - | - | *vdn3* CYP |
| Tv-53368 | TrV_009676 (V) | - | - | - | *vdn3* |
| Tv-60010 | TrV_009677 (V) | - | - | - | - |
| - | TrV_009678 (V) | - | - | - | - |
| Tv-151179 | TrV_009679 (V) | - | - | - | *vdn21* |
| Tv-78733 | TrV_009680 (V) | - | - | - | *vdn22* |
| Tv-216144 | TrV_009681 (V) | - | - | - | *vdn19* CYP |
| Tv-128161 | TrV_009682 (V) | - | - | - | *vdn18*, oxidoreductase |
| Tv-91392 | TrV_009683 (V) | - | - | - | *vdn17* |
| Tv-151341 | TrV_009684 (V) | - | - | - | *vdn10* |
| Tv-135139 | TrV_009685 (V) | - | - | - | *vdn16*, MFS superfamily |
| Tv-70971 | TrV_009686 (V) | - | - | - | *vdn15* |

| | | | | | |
|---|---|---|---|---|---|
| Tv-53581 | TrV_009687 (V) | - | - | - | *vdn14*, glyoxalase/dioxygenase |
| Tv-53582 | TrV_009688 (V) | - | - | - | *vdn13* |
| Tv-151337 | TrV_009689 (V) | - | - | - | *vdn12* CYP |
| Tv-230740 | TrV_009690 (V) | - | - | - | *vdn1* O-methyltransferase |
| Tv-151142 | TrV_009691 (V) | - | - | - | *vdn9* |
| Tv-53690 | TrV_009692 (V) | - | - | - | *vdn8* CYP |
| Tv-151220 | TrV_009693 (V) | - | - | - | *vdn7* dehydrogenase/reductase |
| Tv-78735 | TrV_009694 (V) | - | - | - | *vdn6* |
| Tv-170440 | TrV_009695 (V) | - | - | - | *vdn5* |
| Gliotoxin (GTX)-BGCs | | | | | |
| JGI ID | Gv29-8 | CBS1-2 | P1 | FT-101 | Annotation |
| | SM-BGC 1.1 | SM-BGC 6.4 | | | |
| Tv-216146 | TrV_000002 (I) | - | - | - | *gliA* |
| Tv-83751 | TrV_000003 (I) | - | - | - | |
| Tv-83751 | TrV_000004 (I) | - | - | - | |
| Tv-216149 | TrV_000005 (I) | - | - | - | |
| Tv-138628 | TrV_000006 (I) | - | - | - | *gliT* |
| Tv-216138 | TrV_000007 (I) | - | - | - | *gliH* |
| Tv-78708 | TrV_000008 (I) | TRC2_008324 (VI) | - | - | *gliP* |
| Tv-216161 | TrV_000009 (I) | TRC2_008325 (VI) | - | - | *gliC* |
| Tv-91355 | TrV_000010 (I) | - | - | - | *gliN* |
| Tv-151379 | TrV_000011 (I) | TRC2_008323 (VI) | - | - | *gliK* |
| Tv-53497 | TrV_000012 (I) | TRC2_008326 (VI) | - | - | *gliI* |
| Tv-216157 | TrV_000013 (I) | TRC2_008321 (VI) | - | - | *gliG* |
| Tv-91346 | TrV_000014 (I) | - | - | - | *gliF* |
| Tv-216154 | TrV_000015 (I) | TRC2_008316 (VI) | - | - | *gliM* |
| Tv-159420 | TrV_000016 (I) | - | - | - | |
| Tv-201436 | TrV_000017 (I) | - | - | - | |
| Tv-216163 | TrV_000018 (I) | TRC2_008322 (VI) | - | - | *gliJ* |

Table S7. Proteomic identification of proteins in culture filtrates

| Strains | CBS1-2 | FT-333 | Gv29-8 | FT-101 | P1 |
|---|---|---|---|---|---|
| Total proteins | 8 | 74 | 98 | 21 | 37 |
| SP proteins | 6 | 30 | 34 | 17 | 29 |
| CAZymes | 3 | 14 | 17 | 9 | 17 |
| Oxidoreductases | 1 | 21 | 21 | 0 | 6 |
| Proteases | 0 | 16 | 16 | 3 | 11 |
| Catalytic activity | 2 | 16 | 15 | 2 | 3 |
| Transferases | 0 | 1 | 0 | 1 | 0 |
| Lysases | 0 | 2 | 1 | 0 | 0 |

Table S8. The number of protein-encoding genes transcriptionally upregulated upon interaction of Gv29-8 and maize seedlings (3,4)

| | The overall gene number in the Gv29-8 genome (12006 protein-encoding genes) | FPRG (365) | AFCG (2082) |
|---|---|---|---|
| Signal peptide protein | 1073 | 42 | 349 |
| CAZyme | 425 | 14 | 158 |
| Protease | 415 | 13 | 100 |
| Membrane protein | 2528 | 75 | 433 |
| TFs | 560 | 1 | 87 |
| SM-BGC 1.1$^{GTX}$ | 11 | | 10 |
| SM-BGC 1.3 | 16 | 1 | 6 |
| SM-BGC 1.4 | 13 | | 3 |
| SM-BGC 1.5 | 12 | | 4 |
| SM-BGC 1.6 | 16 | | 5 |
| SM-BGC 1.7 | 13 | 2 | 4 |
| SM-BGC 1.8 | 10 | | 3 |
| SM-BGC 1.9 | 6 | | 3 |
| SM-BGC 1.10 | 21 | 1 | 8 |
| SM-BGC 1.12 | 12 | | 2 |
| SM-BGC 1.14 | 14 | 2 | 1 |
| SM-BGC 2.1 | 9 | | 4 |
| SM-BGC 2.2 | 14 | | 2 |
| SM-BGC 2.3 | 7 | | 3 |
| SM-BGC 2.4 | 6 | | |
| SM-BGC 2.5 | 20 | | 4 |
| SM-BGC 2.6 | 13 | 1 | 2 |
| SM-BGC 2.8 | 13 | | 1 |
| SM-BGC 2.9 | 17 | 5 | 7 |
| SM-BGC 3.1 | 16 | | 3 |
| SM-BGC 3.2 | 11 | 1 | |
| SM-BGC 3.3 | 7 | | 2 |
| SM-BGC 3.4 | 14 | | 3 |
| SM-BGC 3.5 | 8 | | 1 |
| SM-BGC 3.6 | 7 | | |
| SM-BGC 3.7 | 11 | 1 | |
| SM-BGC 3.8 | 17 | | 8 |
| SM-BGC 3.9 | 6 | | 5 |
| SM-BGC 3.10 | 12 | | 1 |
| SM-BGC 3.11 | 9 | | 5 |
| SM-BGC 3.12 | 19 | | 1 |
| SM-BGC 3.13 | 12 | 4 | |
| SM-BGC 4.1 | 12 | | 2 |

| | | | |
|---|---|---|---|
| SM-BGC 4.3 | 15 | | 5 |
| SM-BGC 4.4 | 11 | | 1 |
| SM-BGC 4.5 | 7 | | 1 |
| SM-BGC 5.1 | 15 | | 7 |
| SM-BGC 5.2 | 13 | | |
| SM-BGC 5.3 | 15 | | |
| SM-BGC 5.4 | 9 | | 3 |
| SM-BGC 5.5$^{\text{VIR}}$ | 23 | | |
| SM-BGC 6.1 | 7 | | 3 |
| SM-BGC 6.2 | 15 | | 4 |
| SM-BGC 6.3 | 15 | | |
| SM-BGC 6.4$^{\text{SID}}$ | 11 | | |
| SM-BGC 6.5 | 8 | | 2 |
| SM-BGC 6.6 | 11 | | 3 |
| SM-BGC 6.7 | 5 | | |
| SM-BGC 6.8 | 14 | | 2 |
| SM-BGC 6.9 | 7 | | |
| SM-BGC 6.10$^{\text{CGP}}$ | 15 | | 7 |
| SM-BGC 6.11 | 11 | | 6 |
| SM-BGC 7.1 | 14 | 2 | 1 |
| SM-BGC 7.2 | 1 | | |
| SM-BGC 7.3 | 8 | | 3 |
| SM-BGC 7.4 | 15 | | 1 |
| SM-BGC 7.5$^{\text{FER}}$ | 16 | 1 | 2 |
| SM-BGC 7.6 | 18 | | |
| SM-BGC 7.7 | 7 | | 4 |
| CAZ-GC 1.1 | 11 | | 5 |
| CAZ-GC 1.2 | 6 | | |
| CAZ-GC 1.3 | 6 | 1 | 3 |
| CAZ-GC 1.4 | 6 | 1 | |
| CAZ-GC 1.5 | 7 | | 2 |
| CAZ-GC 2.1 | 5 | | 1 |
| CAZ-GC 2.2 | 6 | | |
| CAZ-GC 2.3 | 4 | | |
| CAZ-GC 2.4 | 5 | | 2 |
| CAZ-GC 2.5 | 5 | | 2 |
| CAZ-GC 3.1 | 5 | | 2 |
| CAZ-GC 3.2 | 6 | | 3 |
| CAZ-GC 3.3 | 7 | 1 | 2 |
| CAZ-GC 3.4 | 6 | | 2 |
| CAZ-GC 4.1 | 9 | 1 | 1 |

| | | | |
|---|---|---|---|
| CAZ-GC 4.2 | 6 | | 4 |
| CAZ-GC 4.3 | 4 | | 2 |
| CAZ-GC 4.4 | 5 | | |
| CAZ-GC 4.5 | 12 | 1 | 2 |
| CAZ-GC 4.6 | 8 | | 5 |
| CAZ-GC 4.7 | 8 | | 5 |
| CAZ-GC 5.1 | 12 | | 4 |
| CAZ-GC 5.2 | 8 | | 3 |
| CAZ-GC 5.3 | 6 | | 1 |
| CAZ-GC 5.4 | 5 | | 1 |
| CAZ-GC 5.5 | 6 | | 2 |
| CAZ-GC 5.6 | 5 | | 3 |
| CAZ-GC 5.7 | 6 | | |
| CAZ-GC 6.1 | 8 | | 5 |
| CAZ-GC 6.2 | 7 | 1 | 3 |
| CAZ-GC 6.3 | 4 | | |
| CAZ-GC 6.4 | 6 | | 3 |
| CAZ-GC 7.1 | 5 | | |
| CAZ-GC 7.2 | 5 | | 1 |
| CAZ-GC 7.3 | 7 | 1 | 2 |

Table S9. Numbers of evolutionarily conserved fungal-plant interaction genes in five different *Trichoderma* species

| | Gv29-8 | | CBS1-2 | FT-333 | FT-101 | P1 |
|---|---|---|---|---|---|---|
| Complete genome | All protein-encoding genes | 12006 | 8541 | 10740 | 9076 | 9120 |
| | Signal peptide proteins | 1073 | 730 | 981 | 799 | 796 |
| | CAZymes | 425 | 326 | 406 | 351 | 352 |
| | Proteases | 415 | 292 | 393 | 322 | 332 |
| | Membrane proteins | 2528 | 1919 | 2313 | 2022 | 2014 |
| | Transcription factors | 560 | 393 | 509 | 455 | 445 |
| FPRGs | All protein-encoding genes | 365 | 215 | 326 | 244 | 248 |
| | Signal peptide proteins | 42 | 17 | 39 | 25 | 25 |
| | CAZymes | 14 | 7 | 13 | 9 | 10 |
| | Proteases | 13 | 6 | 13 | 10 | 10 |
| | Membrane proteins | 75 | 38 | 63 | 48 | 47 |
| | Transcription factors | 1 | 1 | 1 | 1 | 1 |
| AFCGs | All protein-encoding genes | 2082 | 1192 | 1803 | 1349 | 1375 |
| | Signal peptide proteins | 349 | 243 | 323 | 277 | 268 |
| | CAZymes | 158 | 123 | 151 | 135 | 134 |
| | Proteases | 100 | 62 | 94 | 72 | 76 |
| | Membrane proteins | 433 | 285 | 381 | 300 | 294 |
| | Transcription factors | 87 | 39 | 73 | 55 | 52 |

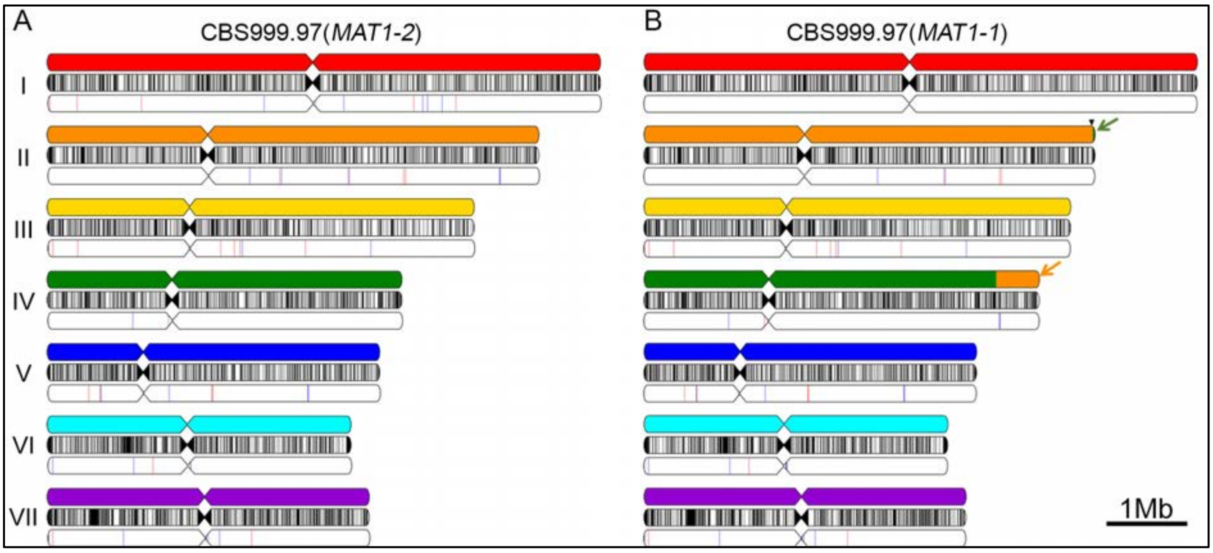


**Fig. S1**. Ideogrammatic representations of the seven telomere-telomere chromosomes of *T. reesei* CBS999.97(*MAT1-2*) and CBS999.97(*MAT1-1*). The colors of chromosome fragments are consistent with their colors in the top CBS1-2 map to clearly show chromosomal rearrangements. Locations of predicted centromeres are shown by restricted width. Locations of AT-rich blocks are indicated by black bars in the middle chromosomal maps. Compared to CBS1-2, the two chromosomal regions in CBS1-1 that underwent reciprocal exchange are indicated by a green arrow and an orange arrow, respectively. The chromosomal location of authentic class I (in red) and class II (in blue) retrotransposons are shown in the bottom chromosomal maps.

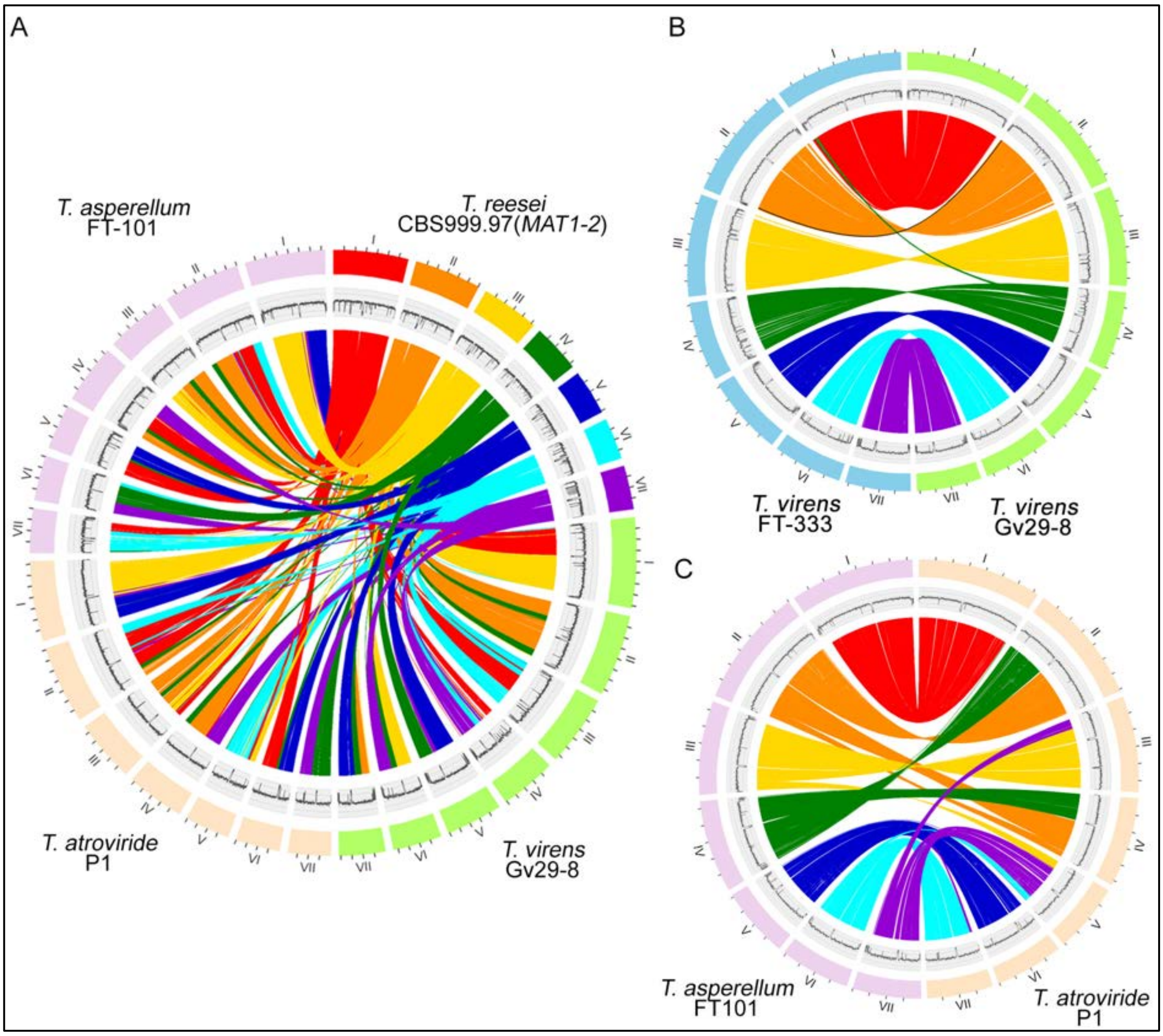


**Fig. S2.** CIRCOS plots of the genomes of indicated *Trichoderma* spp. and their syntenic relationships. The outer circle indicates the seven chromosomes of the near complete genome sequences. The GC contents (window size of 5000 bp) are shown in the middle traces. Chromosome synteny (with ≥10 consecutive protein-encoding genes) is depicted in the inner ribbon tracks of the diagrams. (A) Chromosome synteny between *T. reesei* CBS1-2, *T. asperellum* FT-101, *T. atroviride* P1 and *T. virens* FT-333. (B) Chromosome synteny between *T. virens* FT-333 and *T. virens* Gv29-8. There is a reciprocal translocation event between the first chromosome of FT-333 and the fourth chromosome of Gv29-8. The corresponding chromosomal regions are indicated by a dark green ribbon track in the inner circle of the plot. The right telomeres of the second chromosomes in FT-333 and Gv29-8 contain two NUMTs, which are depicted by a black ribbon track in the inner circle of the plot. (C) Chromosome synteny between *T. asperellum* FT-101 and *T. atroviride* P1. *T. asperellum* and *T. atroviride* are members of Section *Trichoderma.* There are at least six reciprocal translocation events between the genomes of these two biocontrol agents.

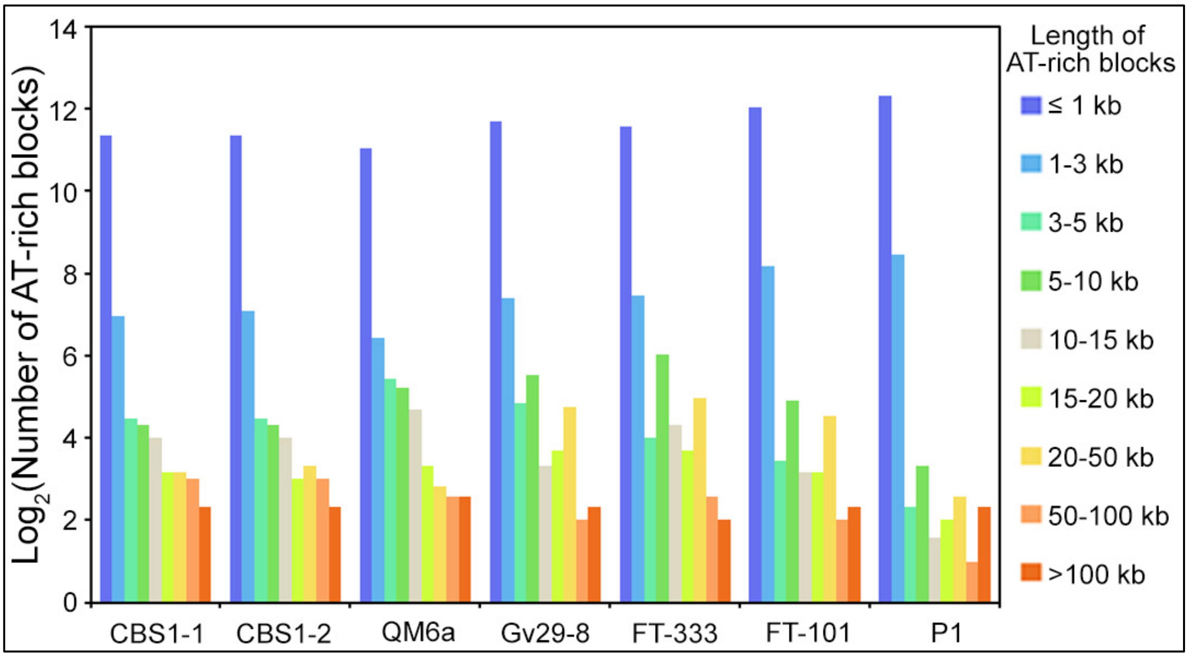


**Fig. S3.** Comparative analysis of the lengths and numbers of AT-rich blocks in the seven *Trichoderma* genomes. Numbers of AT-rich blocks of different lengths are shown.

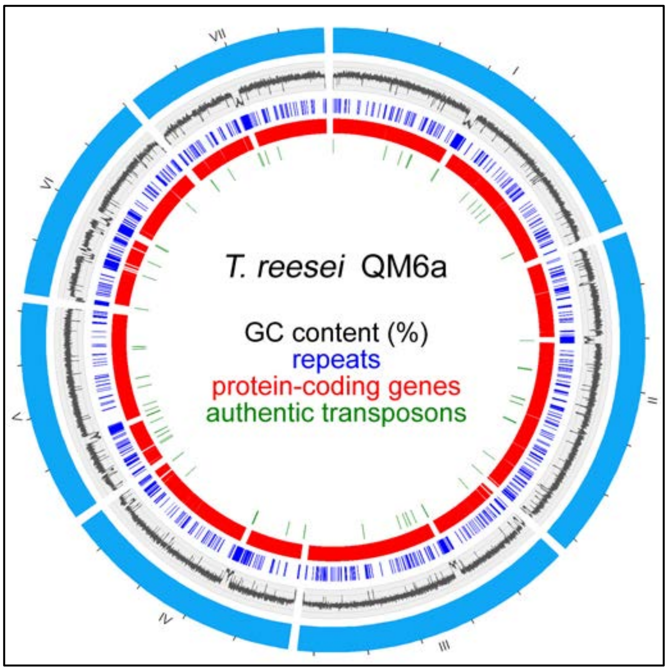


**Fig. S4.** CIRCOS plot of the QM6a genome. The outer circle indicates the seven chromosomes of the near complete genome sequences. The GC contents (%; window size of 500 bp) are shown in the second outer traces. The putative protein-encoding genes (in red) and authentic transposons (in green; Table S2) are shown in the two inner circles, respectively.

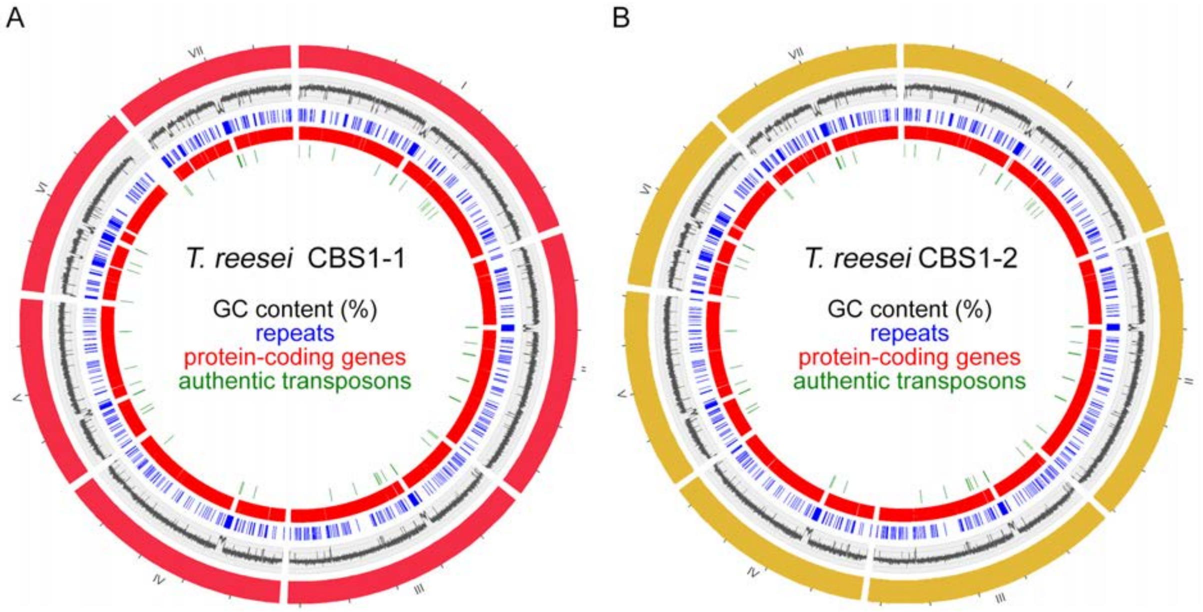


**Fig. S5.** CIRCOS plots of the genomes of CBS999.97(*MAT1-1*) (A) and CBS999.97(*MAT1-2*) (B). The outer circle indicates the seven chromosomes of the near complete genome sequences. The GC contents (%; window size of 500 bp) are shown in the second outer traces. The putative protein-encoding genes (in red) and authentic transposons (in green; Table S2) are shown in the two inner circles, respectively.

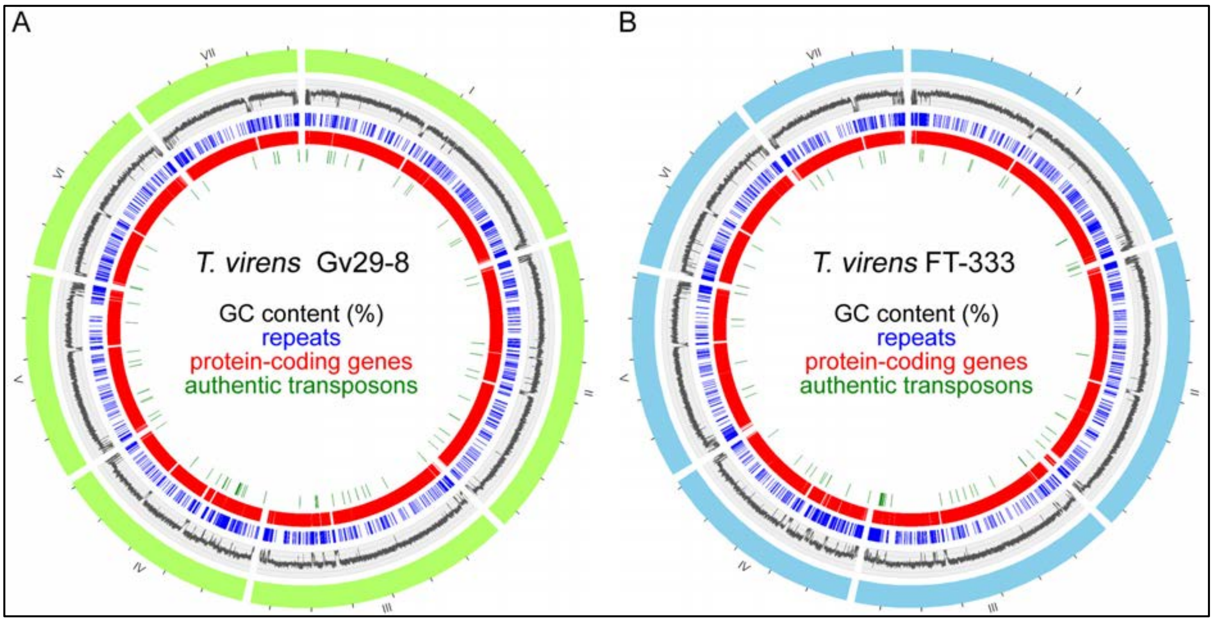


**Fig. S6.** CIRCOS plots of the genomes of Gv29-8 (A) and FT333 (B). The outer circle indicates the seven chromosomes of the near complete genome sequences. The GC contents (%; window size of 500 bp) are shown in the second outer traces. The putative protein-encoding genes (in red) and authentic transposons (in green; Table S2) are shown in the two inner circles, respectively.

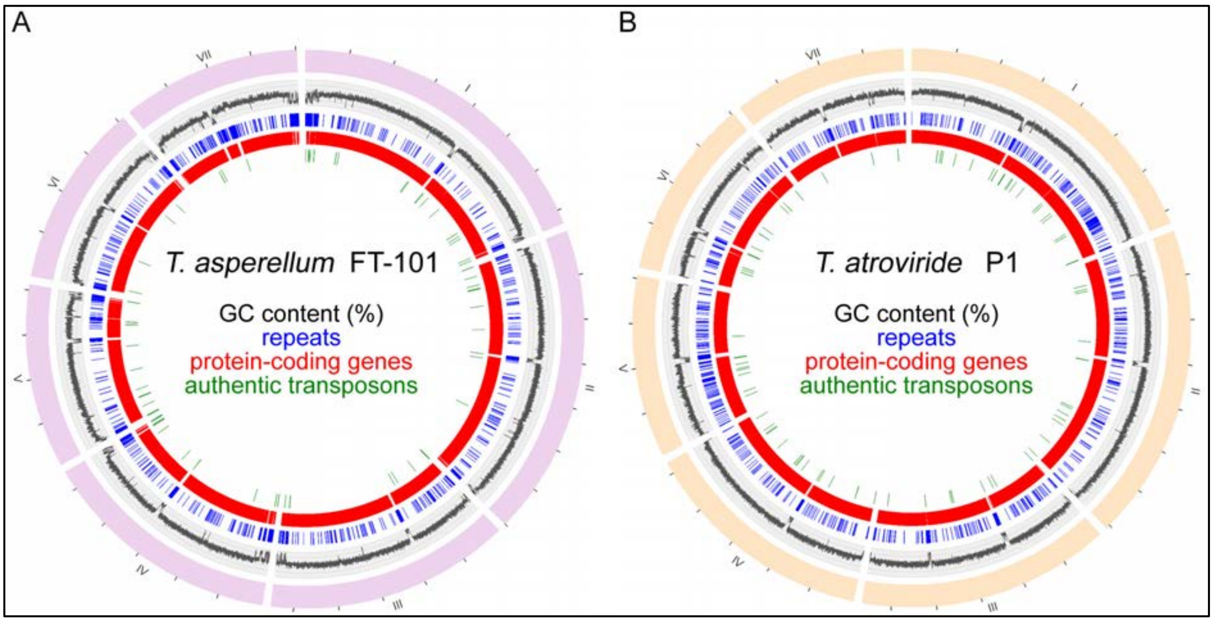


**Fig. S7.** CIRCOS plots of the genomes of FT-101 (A) and P1 (B). The outer circle indicates the seven chromosomes of the near complete genome sequences. The GC contents (%; window size of 500 bp) are shown in the second outer traces. The putative protein-encoding genes (in red) and authentic transposons (in green; Table S2) are shown in the two inner circles, respectively.

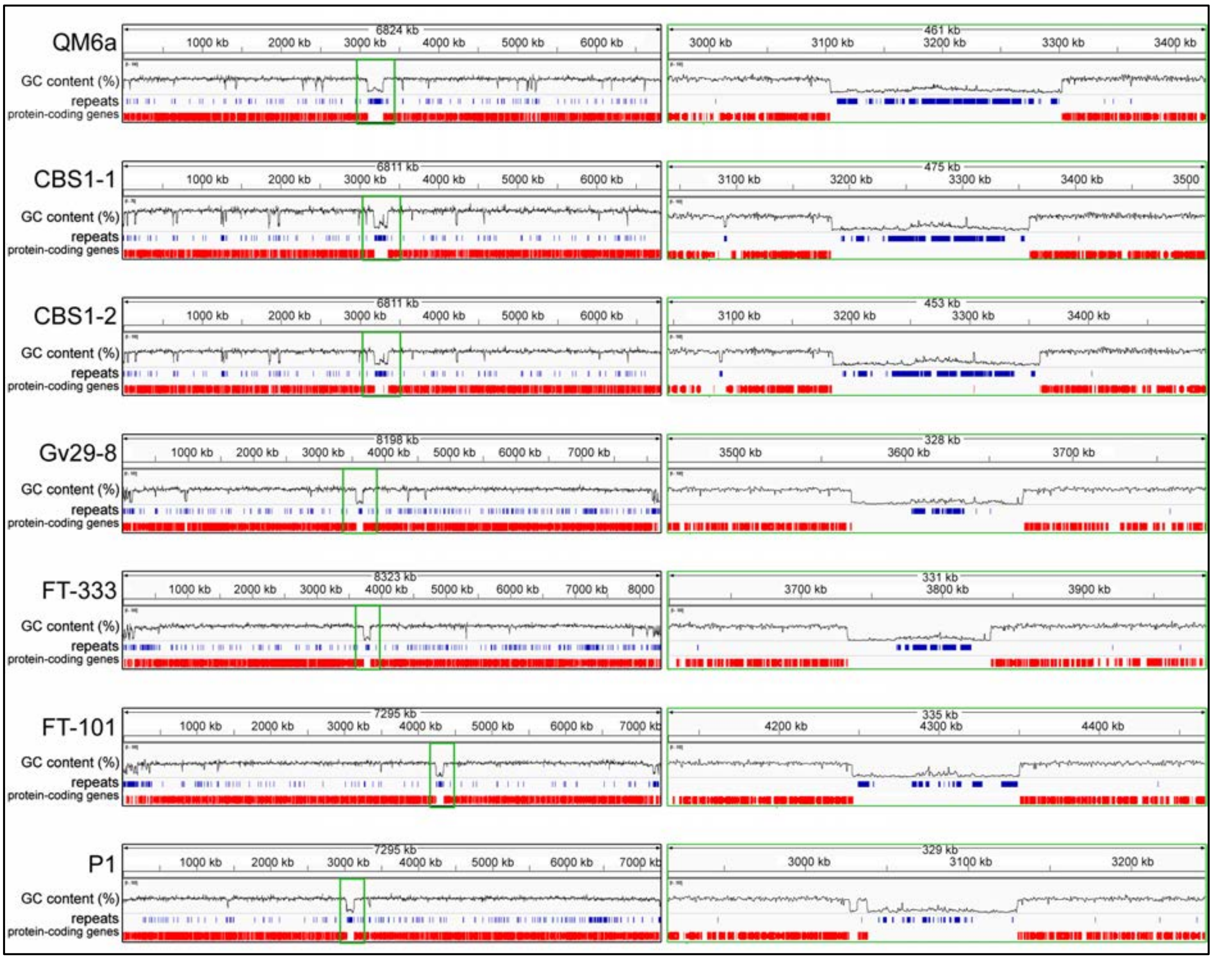


**Fig. S8.** The putative centromeric loci in all seven *Trichoderma* genomes are the longest AT-rich blocks and the longest regions of each chromosome lacking an open-reading frame (ORF) or putative protein-encoding genes. The tracks in the left panels represent graphs of GC content (%) for the first chromosomes of the seven different *Trichoderma* genomes. The tracks in the right panels show the first centromeres (*cen1*) and the corresponding pericentromeric regions. Repetitive sequences are indicated by blue bars, and the putative protein-encoding genes are indicated by red bars.

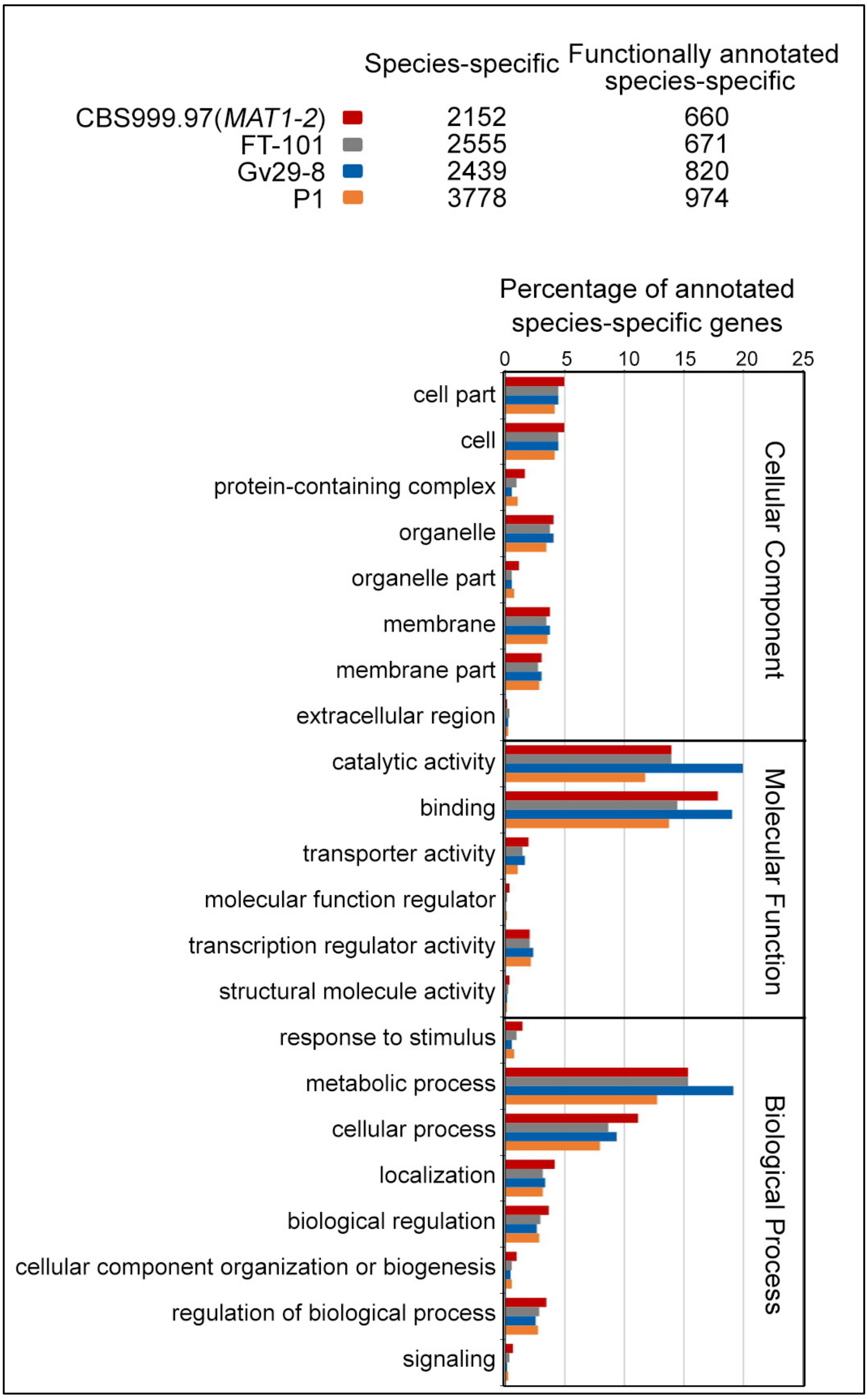


**Fig. S9.** The numbers of species-specific genes that were functionally annotated by the Gene Ontology (GO) annotation software. GO terms are represented as general function categories.

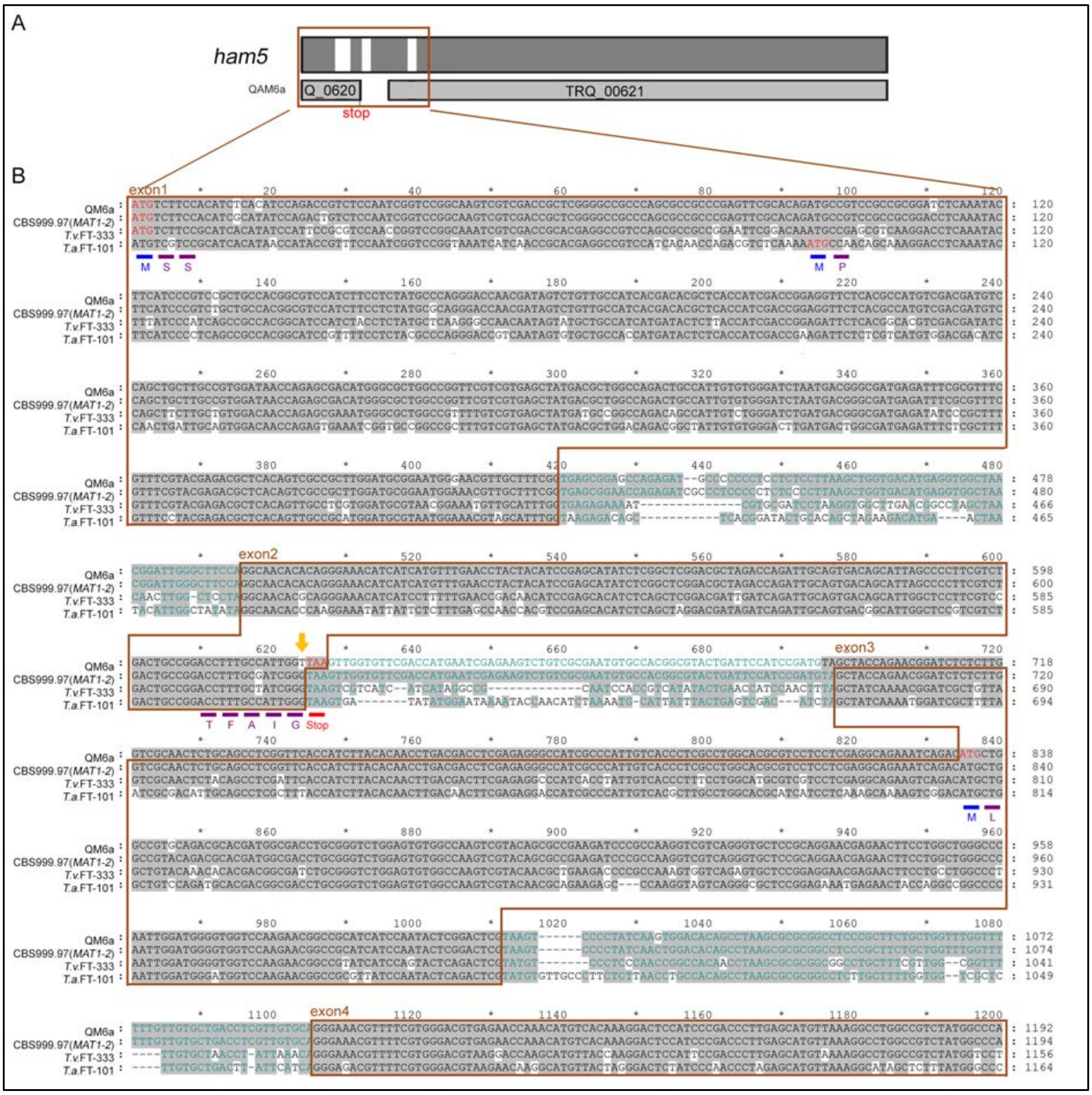


**Fig. S10.** QM6a encodes a defective HAM5 protein. (A) Schematic illustration of the *ham5* gene locus in QM6a. Exons, introns and protein are indicated in dark grey, white, and light grey, respectively. Two putative protein-encoding genes (TrQ_000864 and TrQ_000865) were annotated in QM6a due to a G-to-T point mutation (indicated by a yellow arrow in B). (B) Alignment of the nucleotide sequences of the 5′ portions (exon 1 to exon 4) of the *ham5* genes from four different *Trichoderma* genomes. Nucleotide sequence alignmemt was performed using MAFFT version 7. The translational initiation site (“ATG” in red) of the FT-333 *ham5* gene differs from those of QM6a, CBS1-2 and FT-101.

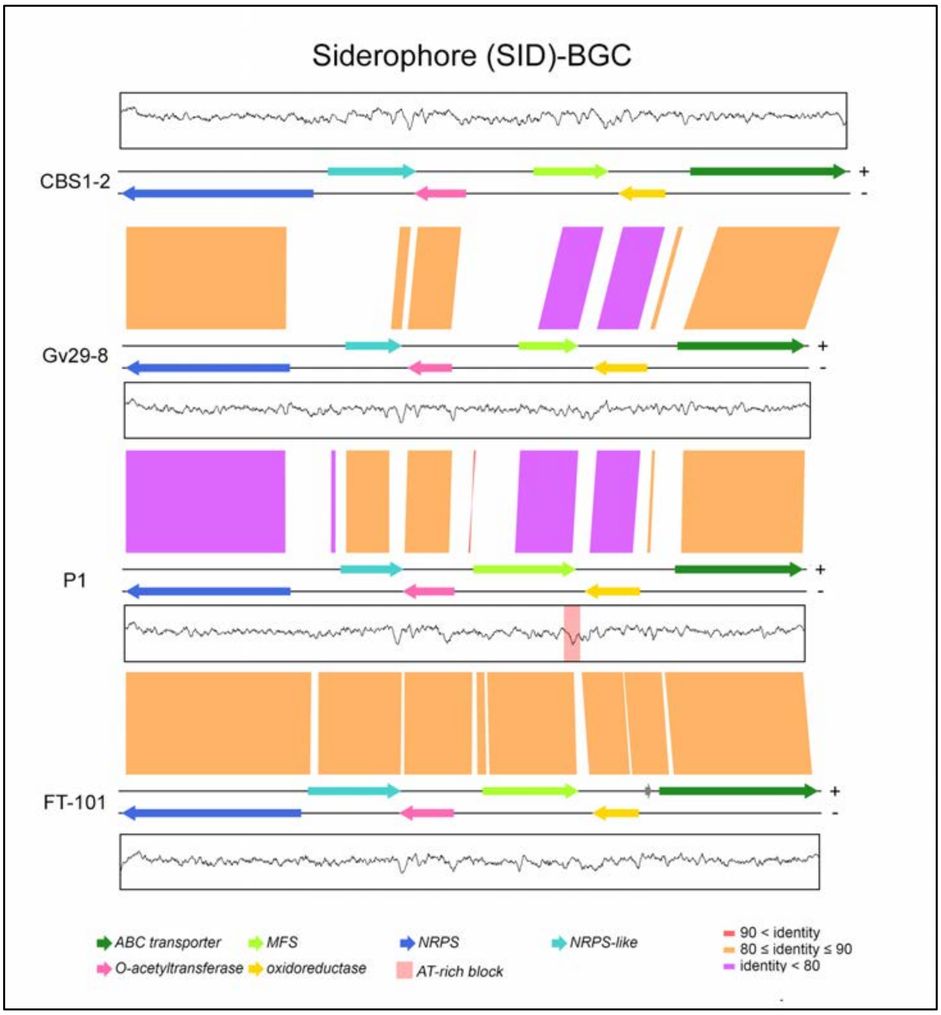


**Fig. S11.** Comparison of the nucleotide sequences within the SID-BGCs in CBS1-2, Gv29-8, P1 and FT-101. The tracks between two strains are color-coded to indicate nucleotide sequence identity. Annotated SM biosynthetic genes and AT-rich blocks are indicated in different colors, respectively.

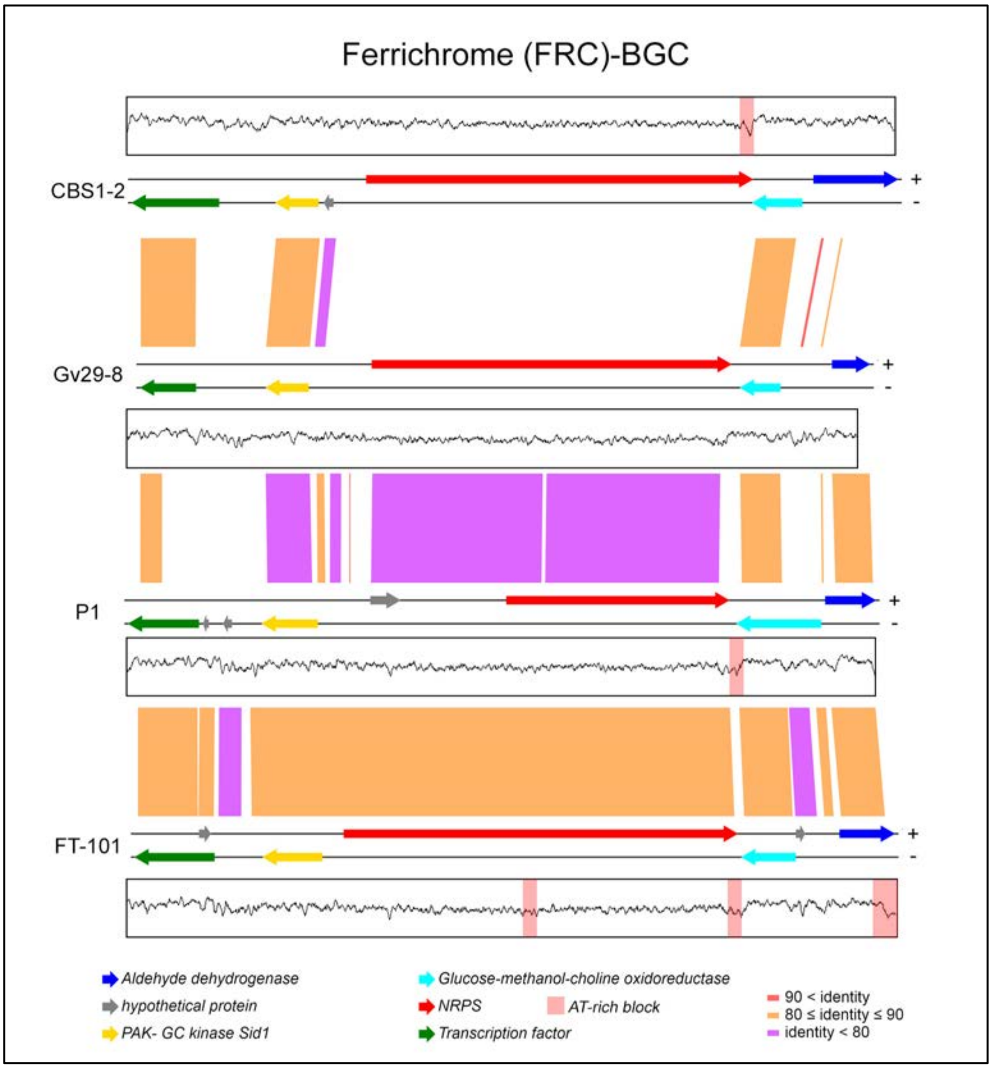


**Fig. S12.** Comparison of the nucleotide sequences within the FRC-BGCs in CBS1-2, Gv29-8, P1 and FT-101. The tracks between two strains are color-coded to indicate nucleotide sequence identity. Annotated SM biosynthetic genes and AT-rich blocks are indicated in different colors, respectively.

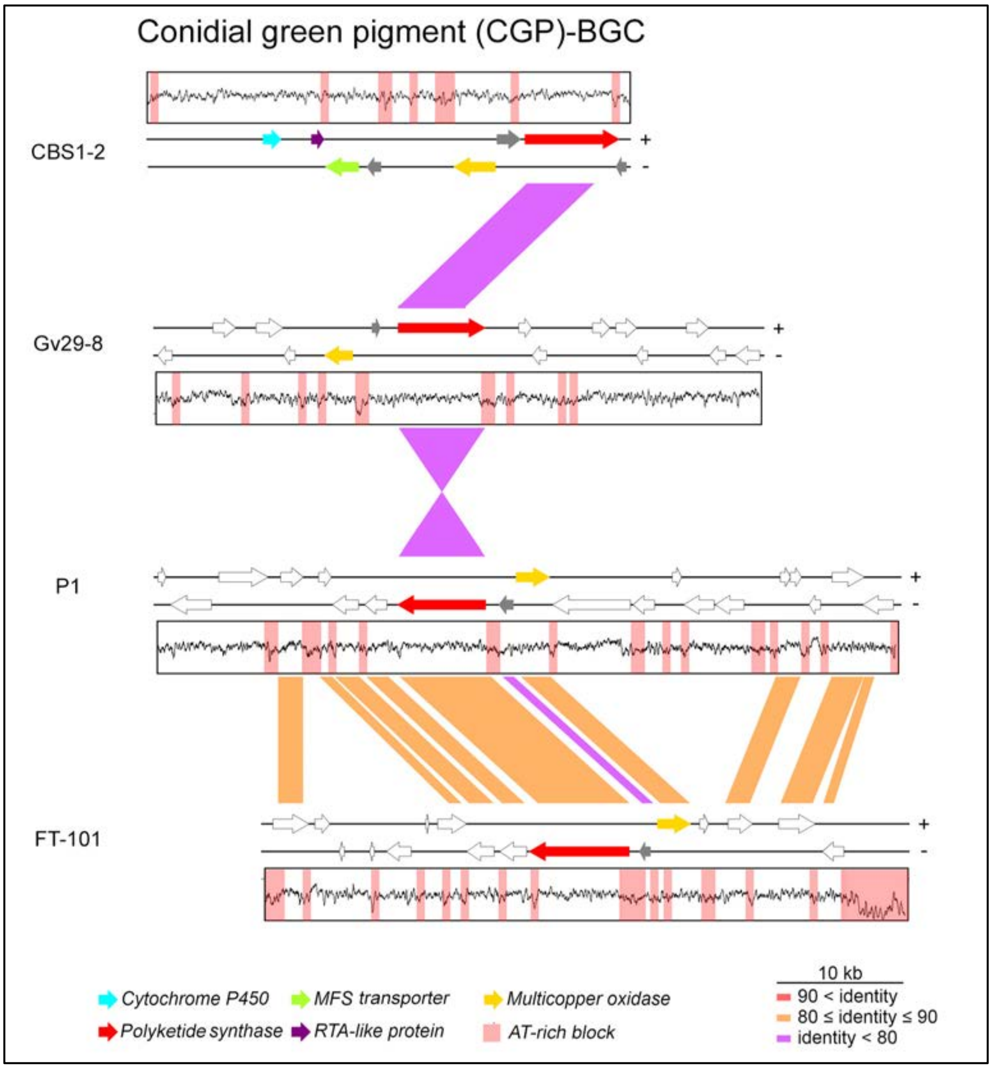


**Fig. S13.** Comparison of the nucleotide sequences within the CGP-BGCs in CBS1-2, Gv29-8, P1 and FT-101. The tracks between two strains are color-coded to indicate nucleotide sequence identity. Annotated SM biosynthetic genes and AT-rich blocks are indicated in different colors, respectively.

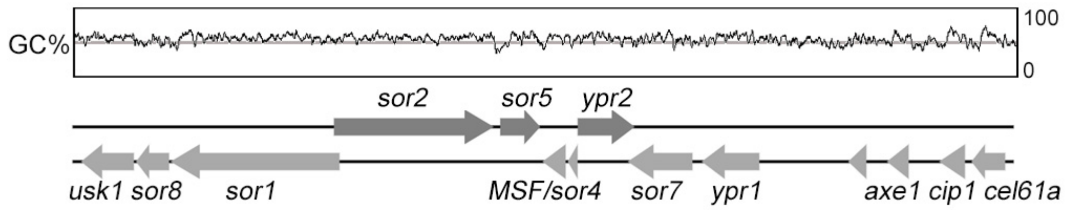


| | QM6a | CBS999.97 (*MAT1-1*) | CBS999.97 (*MAT1-2*) | P1 | FT-101 | Gv29-8 | FT-333 |
|---|---|---|---|---|---|---|---|
| *usk1* | 007352 (V) | 007413 (V) | 007361 (V) | 007655 (IV) | 011012 (VII) | 007389 (IV) | 007148 (IV) |
| *sor8* | 007353 (V) | 007414 (V) | 007362 (V) | 000805 (I) | 001604 (I) | 008886 (V) | 008613 (V) |
| *sor1* | 007354 (V) | 007415 (V) | 007363 (V) | 002470 (I) | 000207 (I) | 006614 (III) | 006373 (III) |
| *sor2* | 007355 (V) | 007416 (V) | 007364 (V) | 008990 (IV) | 010755 (VI) | 002295 (I) | 009185 (V) |
| *sor5* | 007356 (V) | 007417 (V) | 007365 (V) | 003141 (II) | 006848 (IV) | 010936 (VI) | 010612 (VI) |
| *MSF/sor4* | 007357 (V) | 007418 (V) | 007366 (V) | 010536 (V) | 001743 (I) | 006642 (III) | 006401 (III) |
| *sor4_2* | 007358 (V) | 007419 (V) | 007367 (V) | 010536 (V) | 001743 (I) | 006642 (III) | 006401 (III) |
| *sor3/ypr2* | 007359 (V) | 007420 (V) | 007368 (V) | 010535 (V) | 002478 (II) | 006641 (III) | 006400 (III) |
| *sor7* | 007360 (V) | 007421 (V) | 007369 (V) | 011806 (VI) | 005575 (III) | 006377 (III) | 006148 (III) |
| *ypr1* | 007361 (V) | 007422 (V) | 007370 (V) | 008989 (IV) | 012009 (II) | 005971 (III) | 005751 (III) |
| | 007362 (V) | 007423 (V) | 007371 (V) | 000804 (I) | 001605 (I) | 008882 (V) | 008609 (V) |
| *axe1* | 007363 (V) | 007424 (V) | 007372 (V) | 010198 (V) | 011034 (VII) | 006140 (III) | 005918 (III) |
| *cip1* | 007364 (V) | 007425 (V) | 007373 (V) | 005240 (III) | 011951 (VII) | 012230 (VII) | 011859 (VII) |
| *cel61a* | 007365 (V) | 007426 (V) | 007374 (V) | 005241 (III) | 011952 (VII) | 012229 (VII) | 011858 (VII) |

**Fig. S14.** The entire *usk1*-SOR-BGC-*axe1*-*cip1*-*cel61a* chromosomal region encompassing the *T. reesei*-specific SOR-BGC contains 14 protein-encoding genes but no AT-rich blocks. Gene name, gene identities, and chromosome locations (in brackets) of all biosynthetic genes are indicated. The GC content of the chromosomal region in *T. reesei* (window size of 5000 bp) is also shown at top.

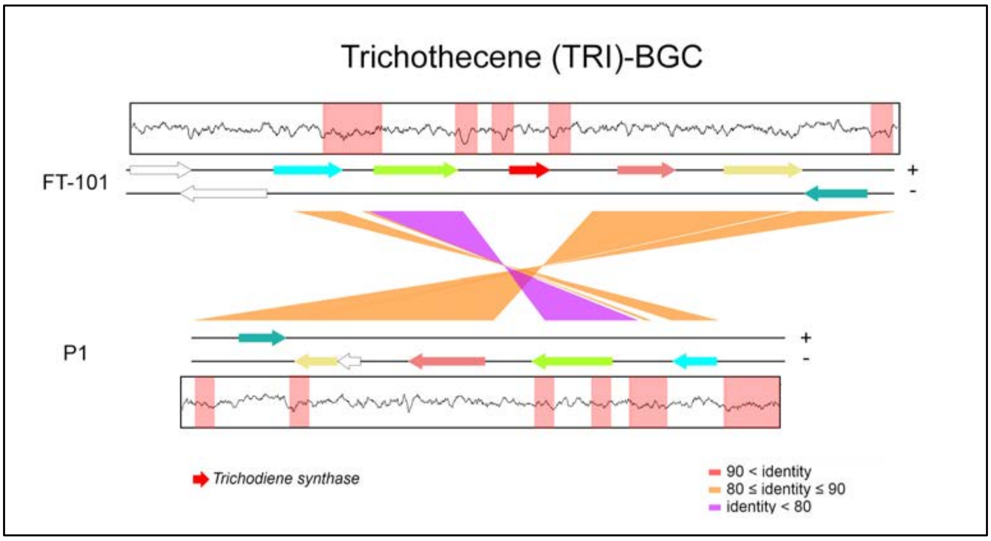


**Fig. S15.** Comparison of the nucleotide sequences within the TRI-BGCs in P1 and FT-101. The tracks between two strains are color-coded to indicate nucleotide sequence identity. Annotated secondary metabolite biosynthetic genes and AT-rich blocks are indicated in different colors, respectively. An ortholog of the trichodiene synthetase *tri5* (red arrow) is absent from P1.

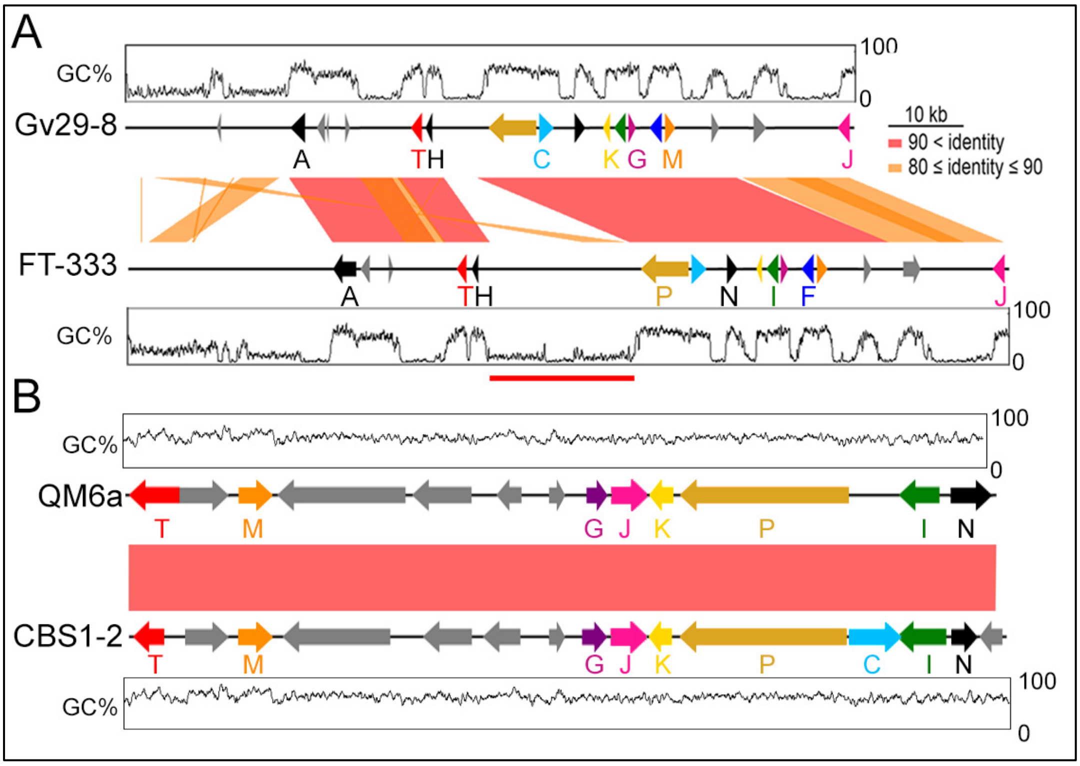


**Fig. S16.** Comparative analysis of GTX- or GTX-like BGCs and secondary metabolites in *T. virens* and *T. reesei*. (A-B) Comparison of the nucleotide sequences within and around GTX- or GTX-like BGCs of Gv29-8 and FT-333 (A), and of QM6a and CBS1-2 (B). Gene name, gene identities, and chromosome locations of all biosynthetic genes are indicated. The GC contents (window size of 5000 bp) are also shown.

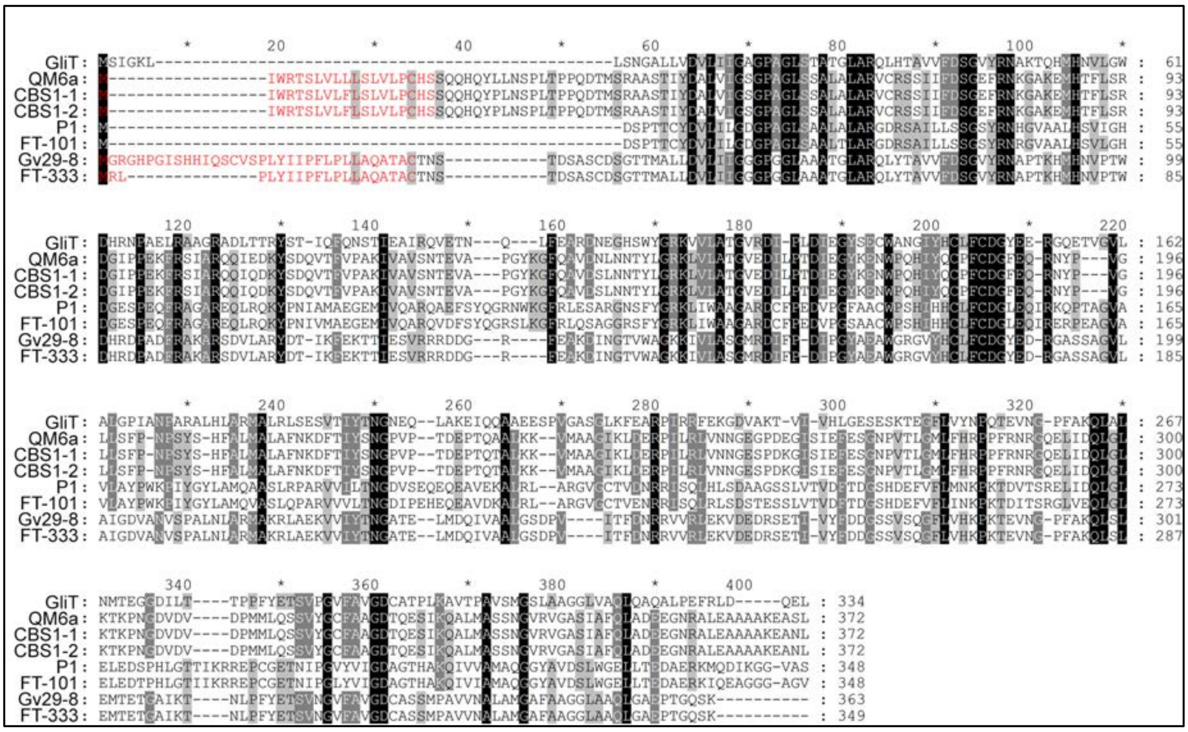

**Fig. S17.** Amino acid sequence alignment of *A. fumigatus* GliT protein (top row) and *Trichoderma* orthologs. The amino acid sequences of signal peptides are indicated in red.

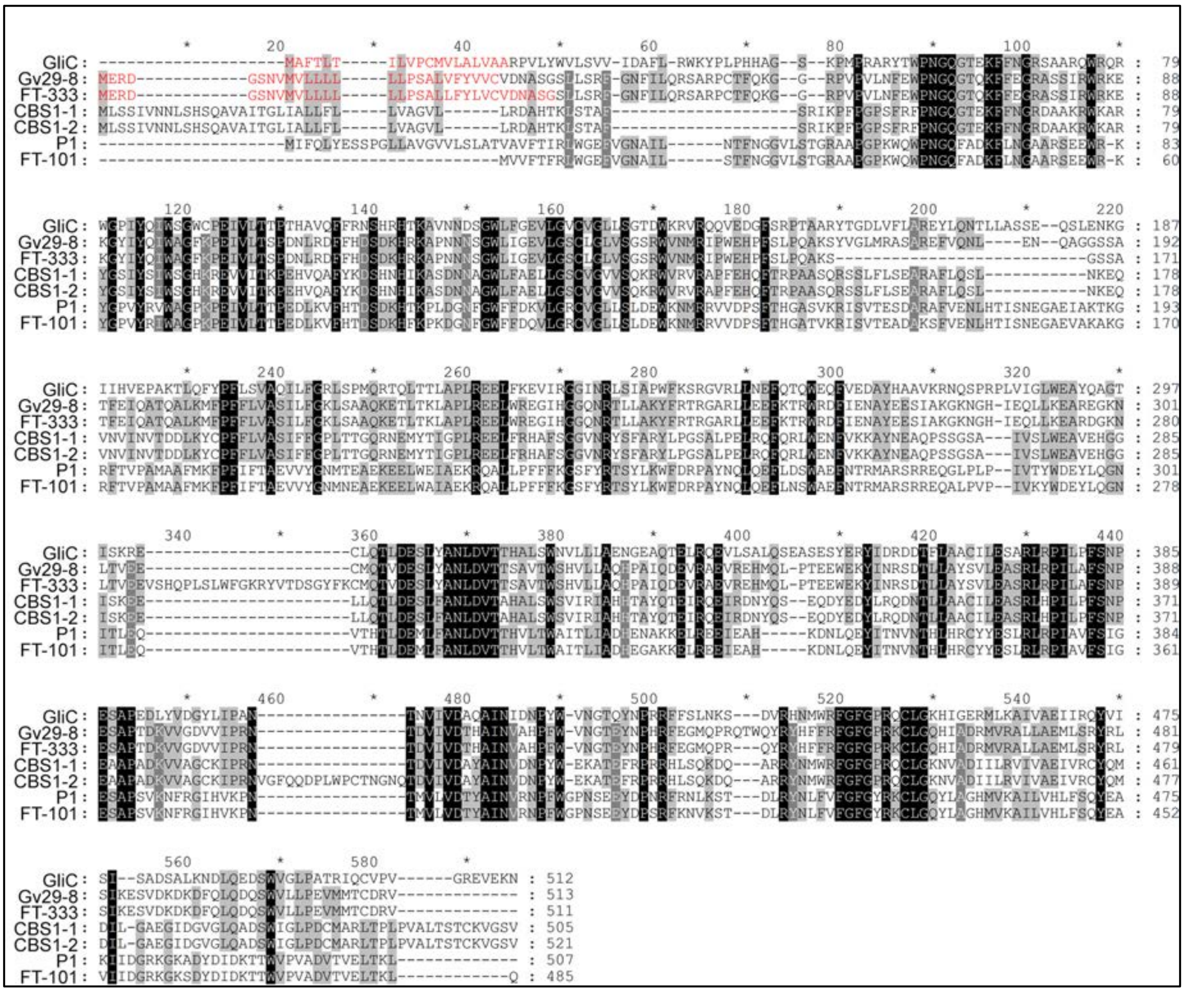

**Fig. S18.** Amino acid sequence alignment of *A. fumigatus* and *Trichoderma* GliC proteins. The amino acid sequences of signal peptides are indicated in red.

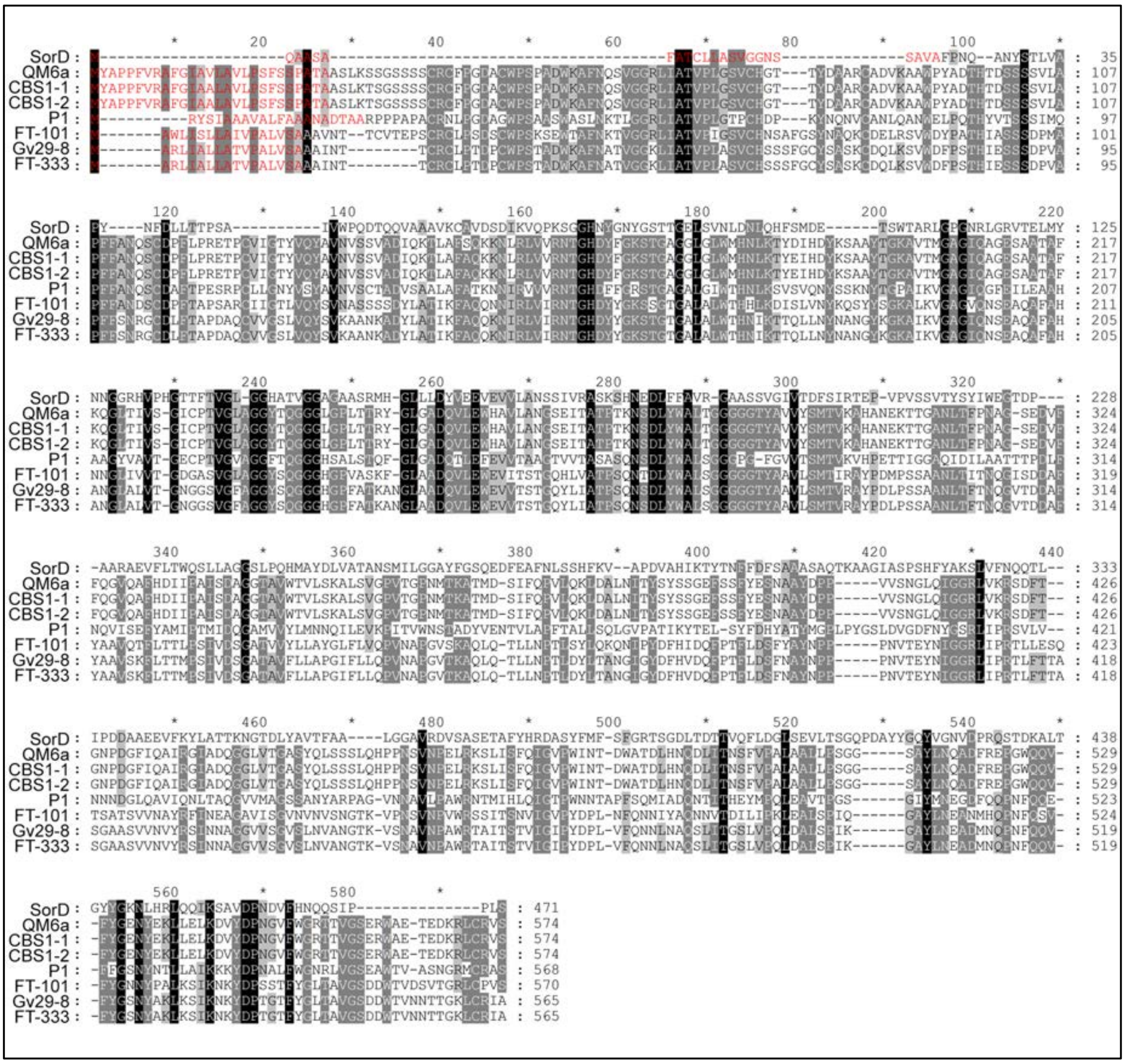

**Fig. S19.** Amino acid sequence alignment of *P. chrysogenum* and *Trichoderma* SorD proteins. The amino acid sequences of signal peptides are indicated in red.

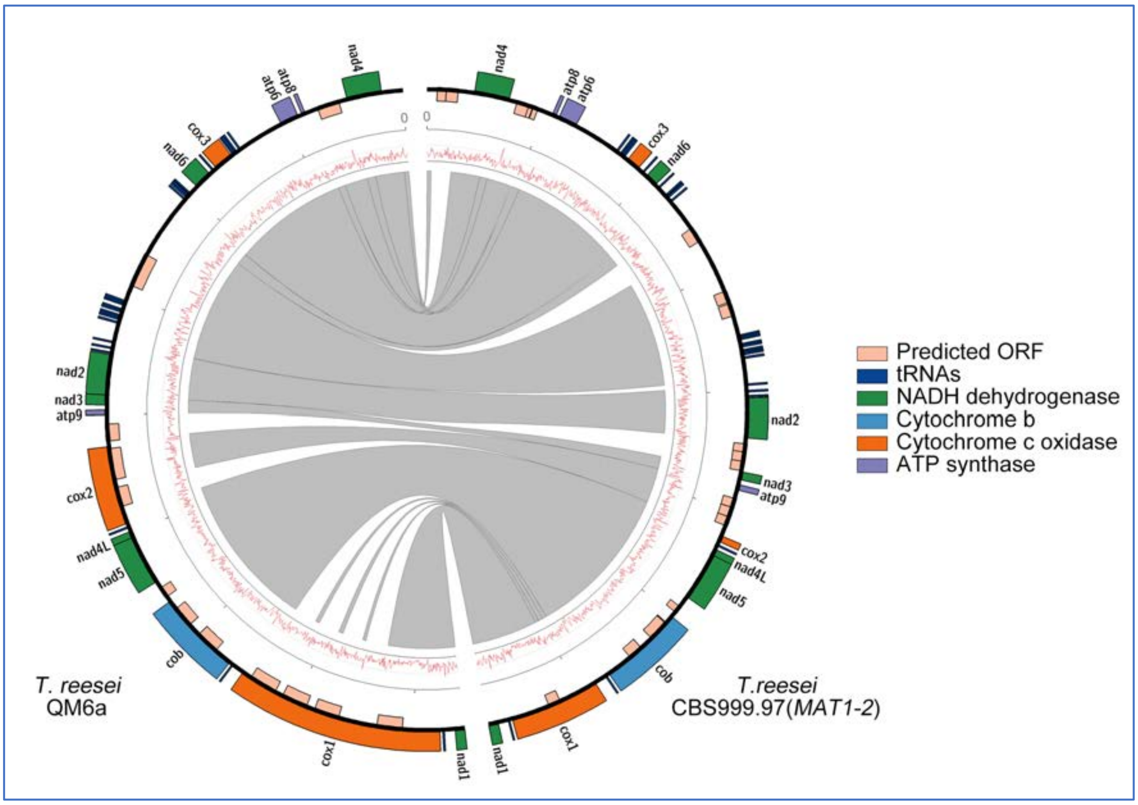


**Fig. S20.** Circular maps of the complete mitogenomes of *T. reesei* QM6a and CBS999.97(*MAT1-2*). The GC contents (window size of 5000 bp) are shown in the middle traces.

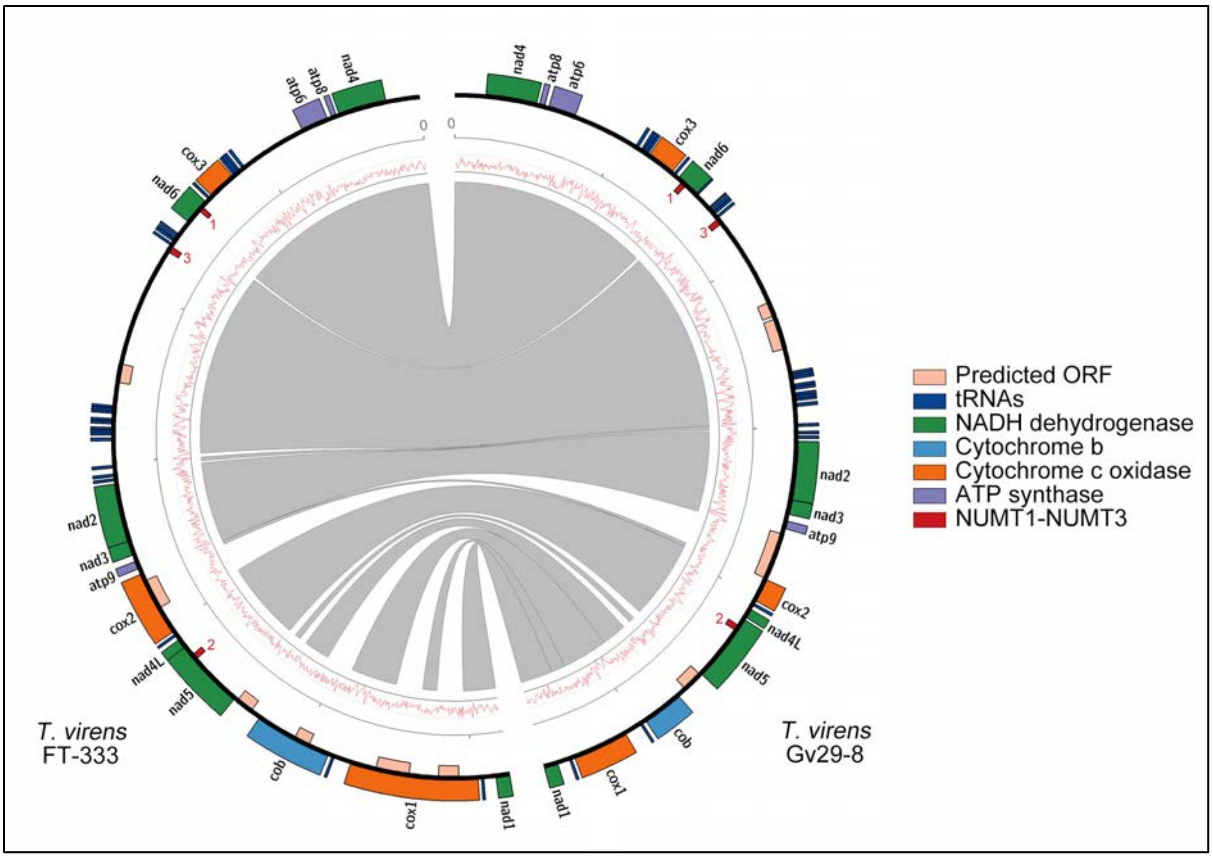


**Fig. S21.** Circular maps of the complete mitogenomes of *T. virens* Gv29.8 and FT-333. The nucleotide sequences homologous to three NUMTs are represented by red blocks. The GC contents (window size of 5000 bp) are shown in the middle traces.

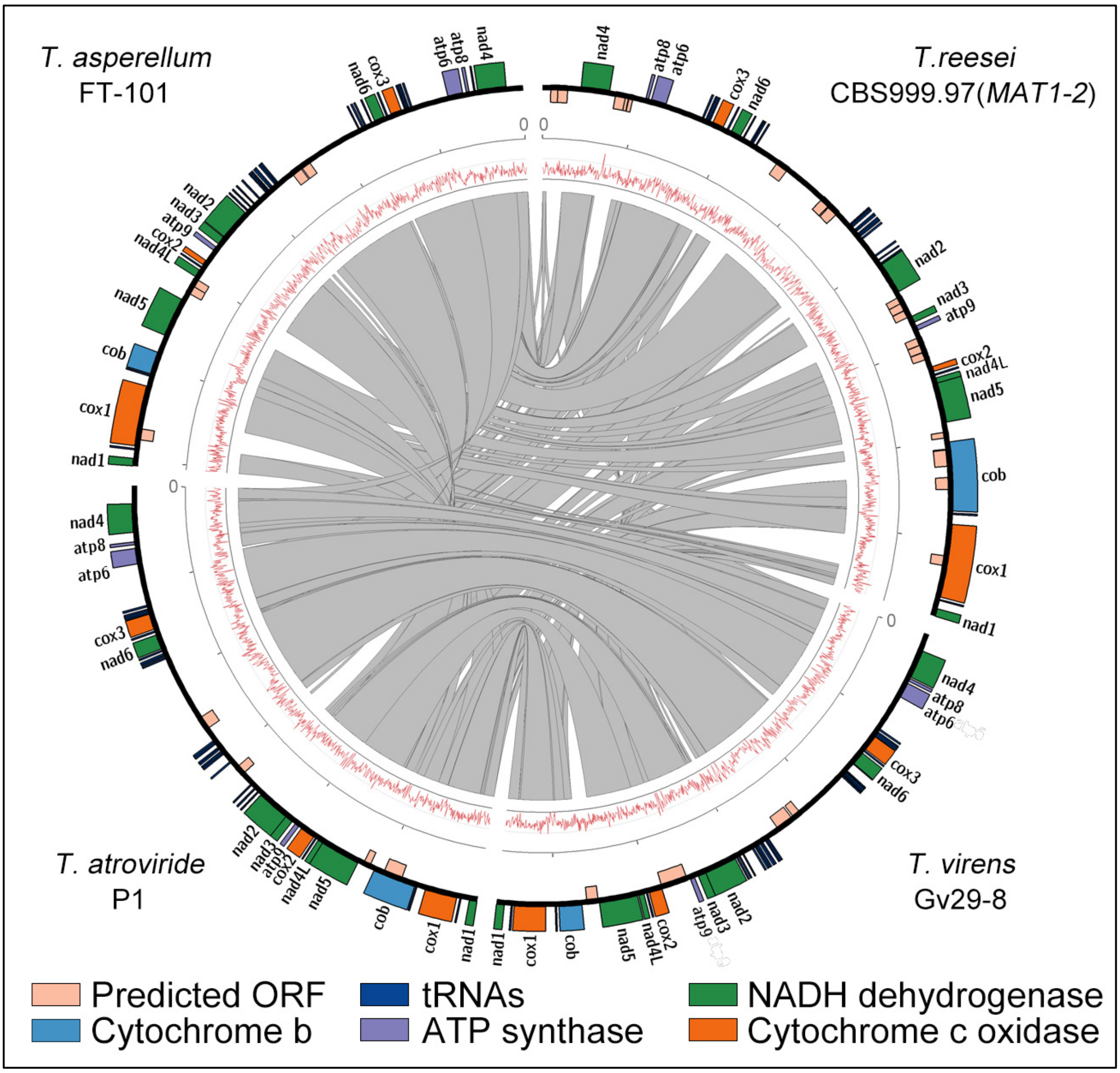


**Fig. S22.** Circular maps of the complete mitogenomes of CBS999.97(*MAT1-2*), Gv29.8. P1 and FT-333. The GC contents (window size of 5000 bp) are shown in the middle traces.

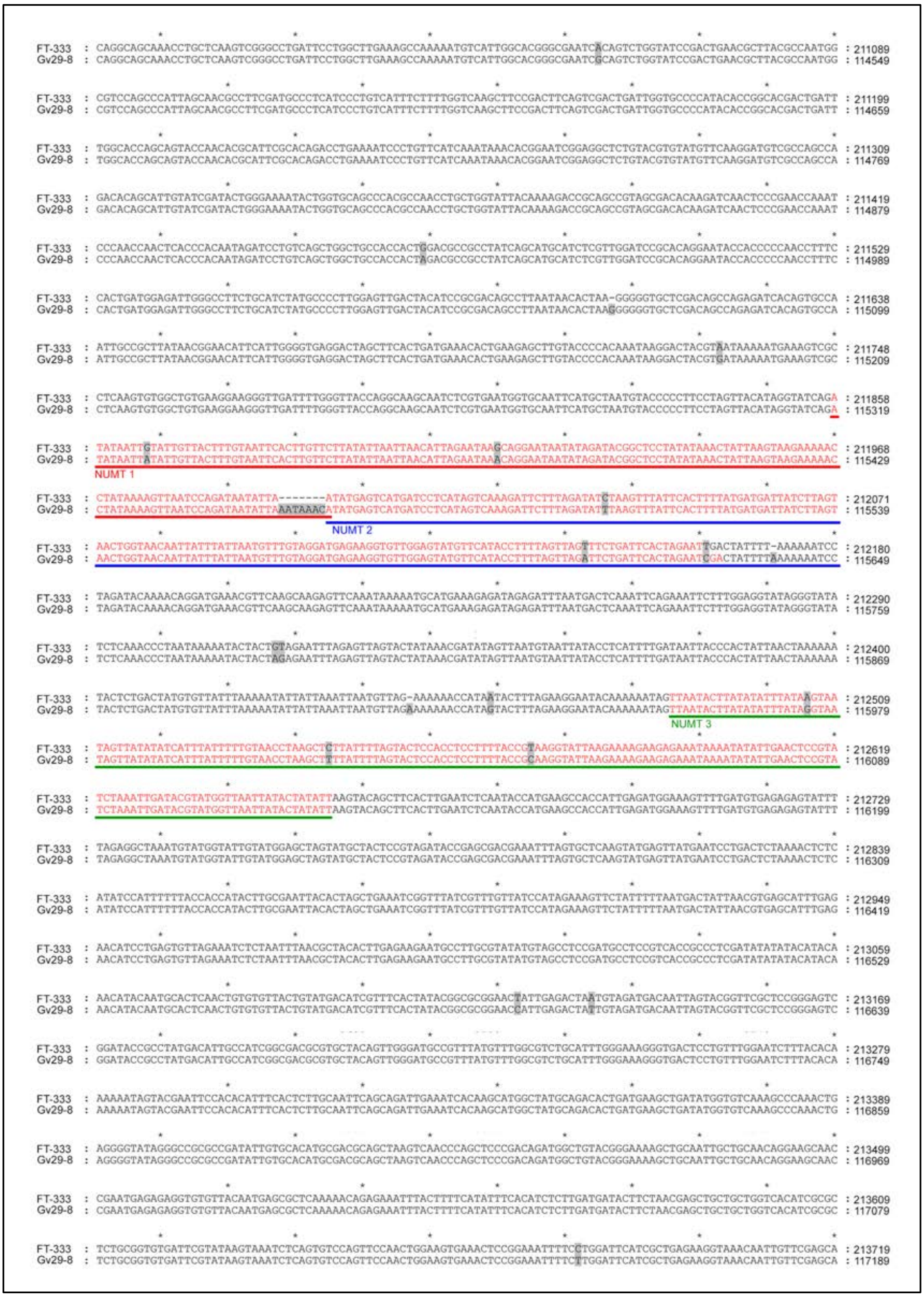


**Fig. S23.** Pairwise sequence alignments of the nucleotide sequences within and around NUMT1 (red bar), NUMT2 (blue bar) and NUMT3 (green bar) in the second chromosomes of Gv29-8 and FT-333. Grey shading highlights differences.

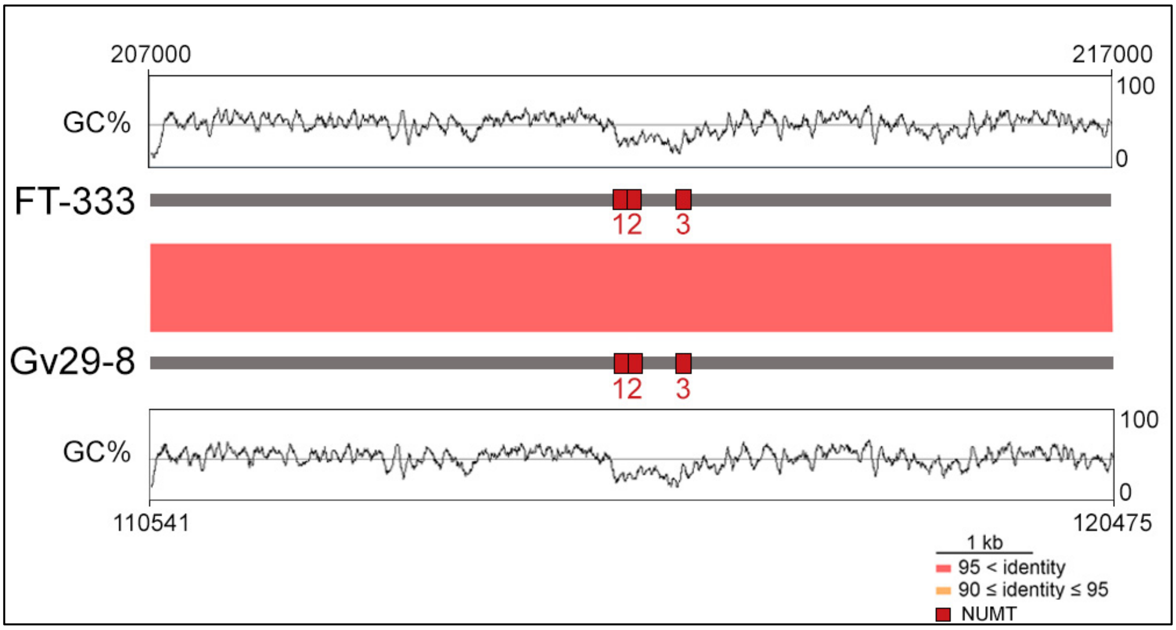


**Fig. S24.** The three NUMTs located in a long AT-rich block. NUMTs are represented by red blocks. The tracks between the two strains are color-coded to indicate nucleotide sequence identity. The GC contents (window size of 120 bp) of the seven chromosomes are shown.

**References:**


1. Li, W.C., Huang, C.H., Chen, C.L., Chuang, Y.C., Tung, S.Y. and Wang, T.F. (2017) *Trichoderma reesei* complete genome sequence, repeat-induced point mutation, and partitioning of CAZyme gene clusters. *Biotechnol Biofuels*, **10**, 170.
2. Li, W.C., Lee, C.Y., Lan, W.H., Woo, T.T., Liu, H.C., Yeh, H.Y., Chang, H.Y., Chuang, Y.C., Chen, C.Y., Chuang, C.N. *et al.* (2021) *Trichoderma reesei* Rad51 tolerates mismatches in hybrid meiosis with diverse genome sequences. *Proc Natl Acad Sci*, **118**, e2007192118.
3. Malinich, E.A., Wang, K., Mukherjee, P.K., Kolomiets, M. and Kenerley, C.M. (2019) Differential expression analysis of *Trichoderma virens* RNA reveals a dynamic transcriptome during colonization of Zea mays roots. *BMC Genomics*, **20**, 280.
4. Wang, K.D., Gorman, Z., Huang, P.C., Kenerley, C.M. and Kolomiets, M.V. (2020) *Trichoderma virens* colonization of maize roots triggers rapid accumulation of 12-oxophytodienoate and two -ketols in leaves as priming agents of induced systemic resistance. *Plant Signal Behav*, **15**, 1792187.
